# Bee Pollen as a Source of Biopharmaceuticals for Neurodegeneration and Cancer Research: A Scoping Review and Translational Prospects

**DOI:** 10.3390/molecules29245893

**Published:** 2024-12-13

**Authors:** Rachid Kacemi, Maria G. Campos

**Affiliations:** 1 Observatory of Drug-Herb Interactions, Faculty of Pharmacy, Heath Sciences Campus, University of Coimbra, Azinhaga de Santa Comba, 3000-548 Coimbra, Portugal; kacemi@gmail.com; 2 Coimbra Chemistry Centre (CQC, FCT Unit 313) (FCTUC), University of Coimbra, Rua Larga, 3004-531 Coimbra, Portugal

**Keywords:** aging, apoptosis, autophagy, bee pollen, cancer, epigenetics, ferroptosis, human microbiota, inflammation, neurodegeneration, oxidative stress, polyphenols

## Abstract

Bee Pollen (BP) has many advantageous properties relying on its multitargeting potential, a new tendency in managing many challenging illnesses. In cancer and neurodegeneration, the multiple effects of BP could be of unequaled importance and need further investigation. Although still limited, available data interestingly spotlights some floral sources with promising activities in line with this investigation. Adopting scoping review methodology, we have identified many crucial bioactivities that are widely recognized to individual BP compounds but remain completely untapped in this valuable bee cocktail. A wide range of these compounds have been recently found to be endowed with great potential in modulating pivotal processes in neurodegeneration and cancer pathophysiology. In addition, some ubiquitous BP compounds have only been recently isolated, while the number of studied BPs remains extremely limited compared to the endless pool of plant species worldwide. We have also elucidated that clinical profits from these promising perspectives are still impeded by challenging hurdles such as limited bioavailability of the studied phytocompounds, diversity and lack of phytochemical standardization of BP, and the difficulty of selective targeting in some pathophysiological mechanisms. We finally present interesting insights to guide future research and pave the way for urgently needed and simplified clinical investigations.

## 1. Introduction

Pharmaceutical drug discovery is facing an overwhelming challenge in many areas of human medicine. Cancer, for example, continues to be one of the most challenging health burdens worldwide. In 2022, there were close to 20 million new diagnosed cases along with 9.7 million deaths from cancer [1]. Neurodegenerative diseases (NDD) are another example of global challenges where disease-modifying therapies are still desperately lacking. This kind of disorders are currently the leading cause of disability and the second foremost cause of death worldwide [2]. Dementia, the obvious manifestation of neurodegeneration, is developing at a frightening pace in the absence of any concrete disease-modifying treatments. While the percentage of aged persons (>60 years) will exceed 21% in 2050 compared to 10% in 2010 [3], the World Health Organization estimates that there are 50 million dementia cases currently in the world with an annual increase rate of 10 million new cases and one new case every 3 s [4]. Cardiovascular diseases are also spreading at an alarming rate in recent years [5], causing at least 18 million deaths worldwide annually [6]. Metabolic diseases are another well-known global epidemic. Diabetes mellitus affects more than 536 million people worldwide, i.e., 10.5% of the world adult population, with an increase rate of more than 36 million persons annually [7]. Obesity is affecting around 14% of the world population (650 million), with 2 billion people being overweight, according to the most recent statistics [8]. In addition, there are at least more than 1000 viruses known to infect humans, and some have killed hundreds of millions of people just in the 20th century [9]. The last pandemic of severe acute respiratory syndrome coronavirus 2 (SARS-CoV-2) infection is just a new example.

In this global health conundrum, bioprospection is taking advantage of a more open world and easy movement of persons, knowledge, and resources to dig in an endless reservoir of natural products that are particularly promising in many complicated pathophysiological processes by exerting multiple actions via multiple targets, as we will see for BP. Plants and their endophytes, for example, are an endless source of phytochemicals that are extremely diverse in occurrence and in pharmacological interest. At a global scale, about 374,000 plant species are known to humanity, and only 6% of them had been investigated for their pharmacological interest, considering that phytochemicals constitute about 70% of all known natural products [10]. Correspondingly, this provides an extremely huge stockpile of bee derivatives, including BP, as we will see hereinafter, which can originate from some of these plants and be potentially used for nutritional, preventive, and curative purposes in humans and animals.

BP is an extremely variable product from biological, chemical, and pharmacological points of view. As we will detail throughout this work, recent studies have shown that BP can modulate many biochemical and physiological processes related to common disorders, including neurodegenerative, cancerous, inflammatory, cardiovascular, and metabolic ones. This product has recently been widely promoted and consumed as a nutritional supplement and functional food due to its richness in valuable nutrients and growing data about its role in ameliorating many physiological and pathological conditions [11]. Its composition can largely vary depending on its botanical origins, even at the subspecies level and generally reflects a mixture of these origins in the same BP sample [12]. Moreover, such composition may vary depending on the bee colony, even in the same apiary, and also depending on the hour, the weekday, or other timing periods [13]. It also varies depending on environmental conditions and geographical origin [14]. All these conditioning factors make BP an extraordinarily rich cocktail and distinguish it from other bee products.

In the context of cancer and neurodegeneration, there is also a huge gap between diagnostic and technological advancements and real-world achievements in conceiving interventional tools to prevent and treat these devastating pathologies. The rarity of these tools in cancers and their quasi-absence in neurodegeneration imply continuous hopes to find new sources of bioactive compounds that are endowed with multitargeting potential in these complex and multifactorial diseases and, at the same time, are verified for an acceptable safety level for prolonged human use. These characteristics perfectly fit the case of BP, as we will develop in our current work.

In our current work, we will firstly give an overview of BP composition with a special focus on its bioactive phytochemicals. This will be a complement to our recent review of its nutrient content [11]. Next, we will elucidate the important potential of this bee-crafted cocktail in pathophysiological alterations that are well-known to be present both in neurodegeneration and cancer pathogenesis. Finally, we will allocate two separate sections to the pathophysiological mechanisms that are specific to neurodegeneration and cancers.

## 2. Phytochemical Overview of Bee Pollen

**Figure 1:**
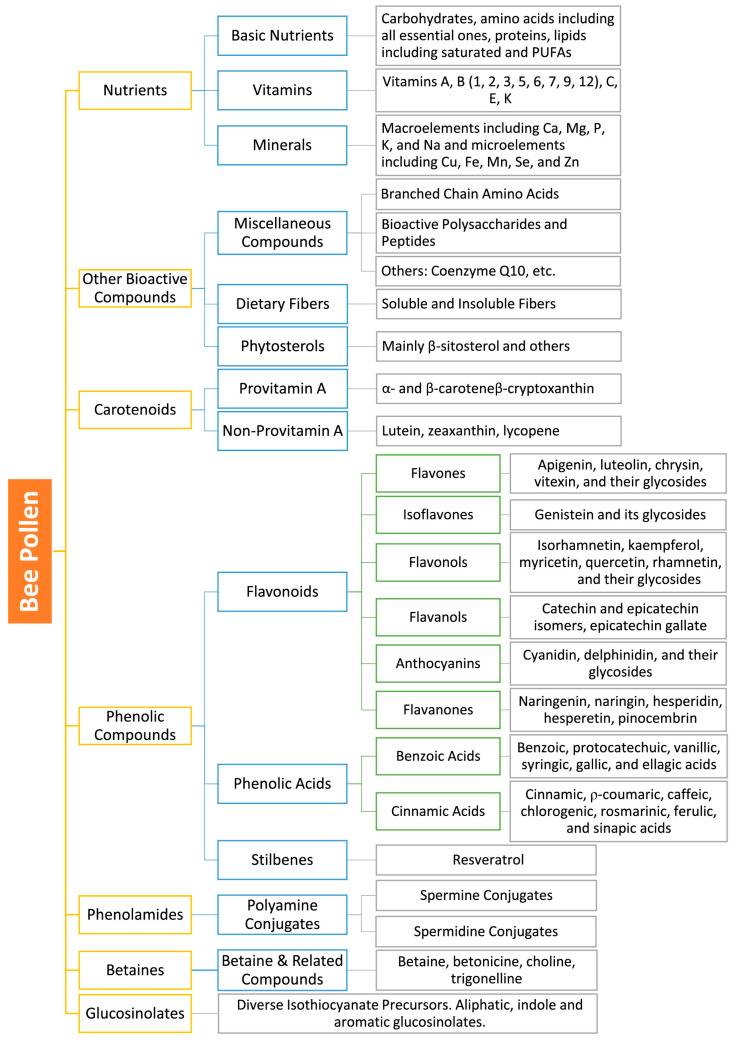
Classification of BP Nutrients and Phytochemicals. Notes: Compounds listed in grey boxes represent major reported compounds and are not exhaustive listings of the corresponding categories. Branched-chain amino acids, which are substantially present in BP [112,113], and many BP polysaccharides and peptides were reported for their significant pharmacological activities in neurodegeneration and/or cancer and will be elucidated through some examples in this review.

**Figure 2:**
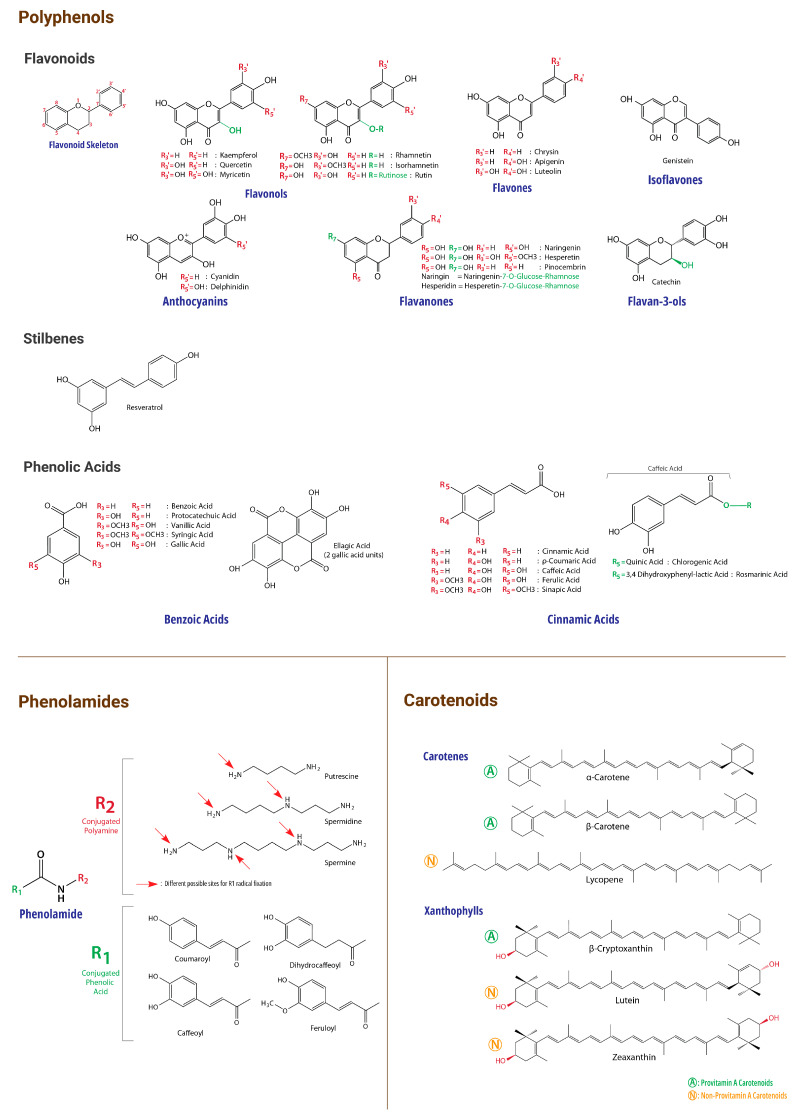
Chemical Structures of Reviewed BP Bioactive Compounds.

In addition to the vast array of nutrients that are present in BP and that we have already detailed in a recent publication [11], this matrix contains a myriad of other phytochemicals manifesting a wide range of biological activities. Indeed, several neurodegeneration-and cancer-related bioactivities of BP were reported by studies from different regions and environmental conditions worldwide. It has been especially reported to exert exceptionally marked antioxidant and anti-inflammatory activities [11,15,16], as well as anti-cancer (anticarcinogenic and anti-mutagenic) [17,18], immunomodulatory, and immunostimulant activities [19,20]. BP has also been reported to possess preventive and ameliorative effects on neurodegeneration [21,22], the overall aging process [18,23], and cell death [24], as well as to promote recovery from chronic diseases and to have chemo preventive properties [25]. These properties, added to its nutritional value, endow BP with many interesting and complementary bioactivities that may be useful in complex and multifactorial diseases such as cancers and NDD. During our current review, we will discuss many key molecules and chemical families in BP that are highly relevant to cancer and neurodegeneration. A summary of these families and their amounts that have been so far reported in BP is presented in Table 1. In addition, numerous vital micronutrients have been highlighted for their potential interest in cancer and neurodegeneration in our current review (see our previous publication for details about their presence in BP [11]). For illustrative purposes, chemical structures and chemical classification of the main bioactive BP compounds are presented in Figure 1 and Figure 2.

**Table 1:** Main Chemical Families and Their Reported Abundance in BP ^(1)^.

Chemical (Sub)Family	Evaluated Matrix	Concentration Value or Range	BP Botanical Origin	Country of Origin	References
Total Polyphenols ^(2)^	Raw Dry BP	20–30 mg GAE/g	Numerous monofloral and multifloral BPs	Many from Asia and Europe	[26,27,28,29,30,31]
		30–40 mg GAE/g	Two multifloral BPs	Türkiye	[26]
			ND	Poland	[32]
			BP from *Brassica campestris*	China	[33]
		40–50 mg GAE/g	Multifloral or monofloral from *Castanea sativa*	Türkiye	[26]
	Fresh BP	20–30 mg GAE/g	Monofloral from *Castanea sativa* or *Hedera helix*	Italy	[34]
		30–40 mg GAE/g	BP from *Castanea* sp. (100%) [35]	Portugal	[35]
Flavonoids ^(2)^	Raw Dry BPs	16.13–35.04 mg QE/g	Multifloral pot pollen from stingless bees (*Tetragonula biroi*)	Philippines	[30]
		31.59 mg/g	BP from *Ranunculus* spp.	Finland	[36]
		11.03–18.81 mg QE/g	Monofloral and multifloral BPs	Romania	[22]
		12.28–15.61 mg QE/g	Monofloral and multifloral BPs	India	[27]
		3.26–11.89 mg QE/g	Diverse multifloral BPs	Türkiye	[29]
		11.77 mg QE/g	Multifloral sample	Poland	[32]
		11.09 mg QE/g	BP from *Clematis vitalba*	Hungary	[31]
	BP Extracts	79.21 mg QE/g	Multifloral sample	Türkiye	[37]
		6.74–104.13 mg QE/g	Dichloromethane partitions from diverse monofloral BPs	Thailand	[38]
Anthocyanins (monomeric)	BP Extract	0.19–0.74 mg CGE/g	Multifloral pot pollen from stingless bees (*Tetragonula biroi*)	Philippines	[30]
	Fresh BP	58.16 mg CGE/L ^(3)^	Monofloral BP from *Castanea sativa*	Italy	[18]
	Dry BP	5.16–11.57 mg CE/g ^(4)^	Diverse monofloral BPs	Italy	[39]
	Raw BP	450–800 mg/kg	Blue BPs	Spain	[40]
Phenolic Acids	Dry BP	1.83–6.97mg FAE/g	Many monofloral BPs	Italy	[39]
		15.75–41.95 mg/g ^(5)^	BP from *Brassica, Filipendula, Trifolium*, *and Vicia* spp.	Finland	[36]
Stilbenes	Dry BP	0.156 and 0.193 mg GAE/g	BP from *Trifolium* spp. and *Coriandrum* spp. respectively	Italy	[39]
		2.49 and 3.32 mg/g	BP from *Taraxacum* spp. and *Rubus* spp. respectively	Finland	[36]
Carotenoids	Fresh BP	11.78 mg/kg	Monofloral BP from *Castanea sativa* sp. (88.8%)	Italy	[18]
		12.78–98.62 mg/kg	ND, from diverse geographical locations	Türkiye	[41]
	Dried BP	261.33 mg/kg	Monofloral BP from *Helianthus annuus*	Slovakia	[42]
	Frozen BP	235.17 mg/kg	Monofloral BP from *Helianthus annuus*		
Phenolamides	Fresh BP	Less than 6 mg/g	Monofloral BPs from *Carduus* sp., *Cistaceae* (100%), and *Echium* sp.	Portugal	[35]
		11.5–25.6 mg/g	BPs from *Rubus* sp. (100%) and *Castanea* sp. (100%)		
		39.02 mg/g	*Rosa chinensis* BP	China	[23]
		27.58 mg/g	*Pyrus bretschneideri* BP		
		22.24 mg/g	*Prunus armeniaca* BP		
		19.66 mg/g	*Crataegus bretschneideri* BP		
		More than 15 mg/g	Monofloral BPs from *Brassica rapa*, *Helianthus annuus*, *Actinidia arguta*, and *Prunus salicina*		
	Dry BP	38.7 mg/g	Bi-floral BP: *Crepis capillaris* (59.84%) and *Plantago* sp. (20.14%)	Portugal	[43]
		16.09 mg/g	Monofloral BPs from *Ononis spinosa* and *Astralagus* sp. (˃90%)	Morocco	[44]
Glucosinolates	Dried BP	Up to 1065 mg/kg ^(6)^	*Brassica* spp. BPs were the richest ones	Spain	[45]
Phytosterols	Dried BP	3.153–6.863 mg/g ^(7)^	Multifloral pot pollen from stingless bees (*Tetragonula biroi*)	Philippines	[30]
	Lyophilized BP	9.46 mg/g	*Sorbus aucuparia*	All BP samples were collected by the bumblebee *Bombus terrestris*	[17,18,19,20,21,22,23,24,25,26,27,28,29,30,31,32,33,34,35,36,37,38,39,40,41,42,43,44,45,46]
		7.36 mg/g	*Calluna vulgaris*		
		5.33 mg/g	*Salix caprea*		
		2.47 mg/g	*Cistus* sp.		
		2.46 mg/g	*Cytisus scoparius*		
Betaines	Raw BP	5432–10,104 mg/kg	71 mono- and multi-floral BP samples	Spain	[47]

Abbreviations: CE: cyanidin equivalent; CGE: cyanidin-3-glucoside equivalent; FAE: ferulic acid equivalent; GAE: gallic acid equivalent; ND: botanical source not determined; QE: quercetin equivalent. **Notes:** ^(1)^ Listed results are from the last five years, except the reference [42], for carotenoids which was reported due to the scarcity of data. Unless we specify another bee species, all the examples in which we announce only “BP” are of samples collected by *Apis mellifera*. ^(2)^ In an aim of conciseness, studies that reported total phenolic or flavonoid contents below 20 mg GAE/g and 10 mg QE/g, respectively, and those that reported these contents in mg rutin or mg catechin equivalents were not listed in the table. ^(3)^ Concentrations of anthocyanins were reported in mg CGE/l in an extract that was prepared with 50 mg BP per mLof 95% ethanol ^(4)^ This study indicated that it used cyanidin, not cyanidin-3-glycoside, as the anthocyanin equivalent. ^(5)^ Benzoic acids were only 0.03 mg/g in *Trifolium repens* BP, 0.04 mg/g in *Vicia* spp. BP, and 0.05 mg/g in *Brassica* spp. and *Filipendula* spp. BPs. ^(6)^ The indicated concentration is the sum of the amounts of 15 glucosinolates that were evaluated in studied samples. ^(7)^ Phytosterol levels reported here are comparable to those reported in vegetable oils that are advised as good dietary sources of phytosterols [30].

BP is particularly rich in phenolic compounds (mainly flavonoids and phenolic acids) and carotenoids [11,40,48], but also in widespread and recently identified compounds such as phenolamides, betaines, and others [23,47,49]. Phenolic acids and flavonoids are most likely responsible for a great part of the antioxidant properties of BP [50]. Studies evaluating phenolic content of BP generally report their results with reference to a simple and well-known phenolic compound. The most frequently used measure to assess phenolic function-related potential is the milligram of gallic acid equivalent, which is generally expressed in a gram of BP sample (mg GAE/g). However, a standardization problem arises frequently in experimental studies, as some of them report the “mg GAE/g” value by reference to a gram of the used extract. Some other studies also report this concentration value by reference to the gram of fresh BP. Although most of the studies that we have reviewed reported the “mg GAE/g” value by reference to a gram of raw dry BP, these discrepancies alter the reported results and harden analytical and comparative reviews. The same observation is also correct for total flavonoid content. The latter is generally evaluated in mg of quercetin equivalent (mg QE) in a gram or kilogram of the sample studied. Some rare studies also report their results in mg rutin equivalent. Total phenolic acids are generally not evaluated due to the lack of a standard evaluation method. These assessment methods do not give a clear idea about quantitative composition in the weight of phenolic compounds but permit us to have a good general estimation about the possible potential in bioactivities that are related to phenolic compounds. As a rough overview, the total phenolic content in a gram of raw dry BP ranged habitually around 10–40 mg GAE/g in the studies that have been published in the last five years [22,26,27,28,29,30,31,32,33,34,35,36,37,38,39,51,52,53,54,55,56]. A minimal number of samples were reported to bear amounts of phenolics that are significantly outside this range. The phenolic content in extracts was obviously higher than raw BP. Amounts up to 173.52 mg GAE/g have been reported in an extract of a Turkish multifloral BP collected by *Apis mellifera* [37]. Chinese monofloral BP from *Schisandra chinensis* was also reported to have a phenolic content of 101.83 mg GAE/g [51]. Monofloral *Nelumbo nucifera* BP from China was among the poorest BPs in phenolic compounds (0.37 mg GAE/g) [52]. All these examples are from studies that have been published in the last five years. In older studies, total phenolic content of BP from around the world was reported in recent reviews [53,56], and ranged between 0.69 and 213.20 mg GAE/g in BPs from different botanical and bee species. In these two reviews, total flavonoid content ranged between 0.9 and 77.88 mg QE/g [53] and between 0.1 and 107.00 mg QE/g [56]. These studies did not screen findings based on the matrix in which results were expressed, i.e., BP extract, dry BP, and fresh BP. This is a frequent problem in experimental investigations and may explain the large variation intervals in reported results. Some of the reviewed studies also expressed results in rutin or catechin equivalents.

In addition to phenolics, we will briefly highlight some BP compounds frequently referenced in this work for their relevant bioactivities, which are often not adequately emphasized in BP-related studies.

Carotenoids, for example, are ubiquitous BP compounds (see Table 1 and references [53,57,58]). Some members of this family serve as vitamin A sources in the human body and are referred to as provitamin A carotenoids, with β-carotene as the main representative, along with other precursors such as α-carotene and β-cryptoxanthin [59], which are also found at high levels in some BPs. Non-provitamin A carotenoids include lycopene, lutein, and zeaxanthin [59] and are also frequent in BP. Lycopene is not solely a “tomato marker,” as BP has also been reported to contain significant amounts of this well-studied carotenoid. A recent study reported that lycopene content in fresh and dried tomatoes ranged from 25.4 to 33.5 mg/kg and 701 to 1181 mg/kg, respectively, depending on the harvest period [60]. In certain BPs, lycopene has also been detected in substantial quantities (31.82, 49.67, and 59.18 mg/kg in *Erica* spp., *Castanea sativa*, and a multifloral BPs, respectively [61]).

Phenolamides are a newly reported class of compounds found in BPs. This family of phenolic derivatives, formed by the combination of phenolic acids and primary amines (mono- or polyamines), is widely present in plants and particularly abundant in flowers and pollen grains [62]. Due to their unveiled abundance in BPs, these phytochemicals will markedly increase the value of pollen as functional food and pharmacological pool. A study of 20 BP samples reported that phenolamide percentages exceeded 1% of the total weight in 11 BP samples and even reached 2.8% in pear and 3.9% in rose BPs [23]. Another recent study reported that phenolamide content ranged from 23.1 to 25.6 mg/g in *Castanea* sp. and *Rubus* sp. monofloral BPs, respectively [35]. These levels substantially exceeded those of flavonoid compounds in the samples studied. Phenolamides present a great diversity in their structures, but their bioactivities in the human body remain largely unknown, with antioxidant, anti-inflammatory, neuroprotective, antiallergic, anti-obesity, anti-atherosclerotic, antimicrobial, and antiproliferative activities being the main reported properties from in vitro and in vivo studies [62,63].

Spermidine is a polyamine found in all living cells and supplied to the human body through three main sources: endogenous biosynthesis, food intake, and gut microbial activity [64]. This molecule has been aberrantly marketed as a “revolutionary” lifespan prolonger and neurodegeneration “counteragent”, but “appearances are often deceiving”, as Aesop said centuries ago. Spermidine was reported by many studies to extend lifespan in diverse living organisms and verified to exert many marked activities that are tightly related to neurodegeneration in animal models and human cell lines [64,65,66,67,68]. In clinical trials and other studies in humans, spermidine has also been frequently reported to exert similar effects [69,70,71]. Conversely, a recent clinical trial with 100 participants experiencing subjective cognitive decline found no significant difference in memory performance after 12 months of spermidine supplementation compared to a placebo [72]. Other more appealing results about spermidine safety and dosage have been reported by experimental and clinical studies. For example, high doses of spermidine have been shown to induce oxidative stress and produce toxic aldehydes, potentially leading to cell death in retinal cell cultures and damaging retinal structure and function in animal studies [73]. Another recent population-based study reported that high spermidine plasma levels were associated with pronounced brain aging and served as markers of the onset of Alzheimer’s disease following a mild cognitive impairment [69]. These examples must drive a cautious attitude toward any translation of experimental data, especially in complex and misunderstood disorders.

Polyamines, especially spermidine, have also been widely flaunted for their possible anticancer potential, but special attention should be drawn to these compounds, and further studies are undeniably needed. As we have seen for spermidine in neurodegeneration, excessive levels of polyamines have been reported to exert some pro-oncogenic roles, as these molecules are necessary for the viability of both normal and malignant cells, considering, however, that their interplay in tumor microenvironments and progression is just beginning to be understood and that the targeting of their metabolic pathways is showing promising outcomes in some cancers [74,75,76,77,78].

Betaines have also been recently isolated in large quantities from BPs. A study of BPs from *Brassica napus*, *Cytisus* sp., *Papaver* sp., *Quercus* sp., *Reseda* sp., *Retama* sp., *Rosa* sp., *Rubus* sp., *Teucrium* sp., and *Vicia sativa* reported that betaines were present in notable amounts in all of them. The concentrations of some of the most known betaines in these BPs were 7–4910, 264–52,834, 12–3628, and 13–723 mg/kg BP dry weight for betaine, betonicine, trigonelline, and choline, respectively [47]. As we have seen for phenolamides, these amounts may also exceed those of many flavonoids and other phenolics that were reported in numerous BP samples (see Table 1 for other comparisons). In addition to their anti-inflammatory and antioxidant effects in diverse anatomical locations, betaines, especially betaine and/or choline, were reported to drive a wide range of metabolic and cardiovascular benefits [79,80]. Betaine may also exert some neuroprotective, neurodevelopmental, and anti-neurodegenerative (e.g., ameliorate brain redox homeostasis, excitatory/inhibitory balance, and dendritic and other transmission alterations, and preserve neuronal structure) [79,81,82] and anticancer (reduces experimentally induced tumorigenesis and malignant cell proliferation and is possibly linked to some cancer risk reduction in humans) [80,83,84,85,86,87] effects.

Glucosinolates have also been recently reported to be present in some BPs in substantial amounts and have even been proposed as reliable differentiating biomarkers of BP origin [45,88]. These compounds and some of their derivatives are known to encompass an important anticancer potential [89,90] in addition to a, perhaps less studied but encouraging, potential against NDD [91,92]. However, due to the rarity of the studies that cover their amounts in BP, we will not succinctly review them in our current work.

Anthocyanidins and their glycosides (anthocyanins) are also widely reported flavonoids in BP. Cyanidin, delphinidin, and their 3-glycosides are among the main representatives that are frequently reported in BP [40,56]. Notably, these flavonoid subgroups are widely known for their very diverse bioactivities against cancer [93,94,95] and neurodegeneration [96,97,98].

Some uncommon but notable compounds have also been reported in BP. Genistein, the soybean “mark”, is also the major isoflavone found in BP [99]. Genistein is well-known to exert a pleiotropic series of biological activities that are closely linked to neurodegeneration and cancer, as we will see throughout this work. Another compound of interest is resveratrol, a widely studied and marketed phenolic compound abundant in many plants and recognized for its potential to tackle the aging process and age-related pathophysiological mechanisms. Resveratrol was reported at highly different concentrations in BP samples from distant geographical locations and diverse botanical origins [100,101,102,103,104].

BP is also rich in dietary fibers, although widely variable percentages have been reported in the literature. Recent studies reported percentages from 13 to 22% in most cases, and more rarely 3–10% or 23–31% ranges [56,105]. Old studies reported that dietary fibers were present in BP at percentages ranging from 0.3 to 20% [106]. Some rare studies reported low percentages of dietary fibers in some BPs [56], but this may also be due to methodological issues. In fact, BP-covering layers are mostly composed of polysaccharides, including sporopollenin in the exine and cellulose and pectin in the intine [11,107], and a considerable amount of fibers will thus be surely present, at least due to these polysaccharides. Different extraction media may also obviously result in different dietary fiber yields from the same BP [108].

A mineral that is of particular relevance to carcinogenesis and neurodegeneration pathophysiology is selenium, as we will see. This vital microelement is present in high quantities in many BPs. Surprisingly, ratios up to 3 mg/kg have been reported by a study in Jordanian BP [109] and up to 5 mg/kg in Turkish BP samples [110]. These samples, if the reported results are valid and reproducible, will make these BPs extremely rich sources of selenium. The recommended dietary intake of this vital element will then be largely covered with small quantities of BP. In fact, an expert panel requested by the European Commission have just published an opinion and stated that 255 µg/day should be considered as the tolerable upper intake level of selenium [111].

## 3. Shared Alterations in Neurodegeneration and Cancer

Many pathophysiological traits are known to ubiquitously mark both neurodegeneration and cancer. Some of them are also widely known to be present in other diseases such as cardiovascular and metabolic diseases. Among these shared mechanisms, we will highlight the most importantly investigated ones, i.e., oxidative stress, chronic inflammation, imbalances of cell death and clearance mechanisms, metabolic alterations, functional and compositional alterations of microbiota, and aberrant epigenetic signaling.

### 3.1. Oxidative Stress

Antioxidant activity is one of the most studied properties of BP [43]. The latter has been proven by a great number of studies [11,114] to be a powerful antioxidant with a synergy of numerous antioxidant compounds that it gathers, which are represented mainly by phenolic compounds but also include other diverse phytochemicals. Antioxidative properties that are exerted by BP are evidently present in parallel and complementary ways with its marked anti-inflammatory effect but also participate in the latter [115]. Obviously, antioxidant activities, although generally present regardless of how and where studies were conducted, vary widely depending on the bee and plant species and geographical, soil, timing, and environmental conditions [114,116]. Many experimental and preclinical studies have been published in this context. A review of BP antioxidant studies and unveiled mechanisms can be found in our recent publication [11].

Miscellaneous animal studies have reported that BP dietary supplementation resulted in a significantly higher activation of antioxidant and prophylactic mechanisms in different organs [117,118,119], as well as in ameliorating different pathogenic biochemical, hematological, toxicological, and inflammatory parameters at the systemic level in animals [120,121,122]. Hence, BP has a universal and marked antioxidant potential by acting on major known redox pathways via a spectrum of pleiotropic mechanisms, including radical scavenging, activity modulation of redox enzymes, metal reduction reactions, and cell protection and functional improvement [11]. Although varying depending on their types, BP extracts encompassed antioxidant activities regardless of the used solvent.

Facing such variability, extraction methods will evidently be crucial in determining the antioxidant activity of BP. While ethanolic extracts were repeatedly reported to be more active than other extracts after comparing numerous samples of multifloral BP [116,123], methanolic extract was also reported to be more active than water extract of *Tilia* BP, for example [114]. A recent comprehensive review concluded that 70% aqueous ethanol extraction using the agitation extraction without pulverizing the BP extracts provided the optimal condition to maximize antioxidant principles in these extracts [123].

Antioxidant activity of BP has been shown by many studies to depend not only on botanical origin but also on the geographical origin for the similar botanical composition elucidated by palynological studies [54]. A comparative evaluation review has recently concluded that multifloral pollen showed a higher antioxidant activity, evaluated by radical scavenging and metal-reducing potentials, and a richer phenolic composition [29]. The authors of this study also recommended that prior chemical analysis and bioactivity tests would be better parameters than palynological analysis to assess the value of BP.

### 3.2. Inflammation

Chronic inflammation is a key factor in aging and is sustained and triggered in an auto-maintained loop in aging persons, especially in immunosenescent ones and those hosting pathophysiological events linked to neurodegeneration and cancer [124,125]. In particular, chronic low-grade inflammation has been acknowledged as a major pathophysiological event and bidirectional crosstalk effector in neurodegeneration [126,127] and cancers, including carcinogenesis and metastasis phases [128,129].

A large number of basic and preclinical studies have reported diverse and interesting activities of BP against acute and chronic inflammation. BP is particularly promising in tackling chronic low-grade inflammation due to its richness in anti-inflammatory compounds, its high safety, and its long history of human use and acceptance [11]. Indeed, BP has been reported to act on the main pathophysiological mechanisms of chronic inflammation. This has been experimentally illustrated by the downregulation of pro-inflammatory cytokines, diverse inflammatory enzymes, inflammation-mediating protein complexes (e.g., nucleotide oligomerization domain-like receptor family, pyrin domain containing 3 (NLRP3) inflammasome), inflammatory cell flux and activation, and major inflammatory signaling pathways, i.e., nuclear factor kappa B (NF-κB), mitogen-activated protein kinase (MAPK), and nuclear factor-erythroid 2-related factor 2 (Nrf2) pathways. A comprehensive review of the most recent of these reports was completed in our recent publication [11].

Inhibiting inflammatory process initiation and execution may not be the sole necessary intervention to limit inflammation’s deleterious effects. Inflammation resolution is also a vital phase to abolish these effects in the body and restore tissular and inflammatory response homeostasis and is thus recently becoming a novel therapeutic target in chronic inflammatory diseases [130,131]. Its mechanisms, although remaining not well understood, appear to rely on specialized lipidic pro-resolving mediators (SPMs, which are all derived from omega-3 and omega-6 fatty acids), other molecules, and immune cells, and culminate in regenerating intact endothelial, vascular, and tissular states [130,131]. The failure of these critical processes is likely a key driver behind the settlement of chronic inflammation and resulting chronic diseases, including cancer [132] and NDD [133].

Many natural compounds were reported to accelerate inflammation resolution, including some BP compounds. The main SPM source molecules, viz. omega-3 fatty acids, are present in BP as we have seen. In addition, anthocyanins, some of the most ubiquitous polyphenolic components of BP from different bee species [18,30,40,134], were reported to be important promoters of inflammation resolution [135,136]. Note that anthocyanins are also well studied for their antioxidant potential and interesting inhibitory effects on chronic low-grade inflammation mechanisms [137].

Polarizing macrophages to the M2 phenotype is known to exert an anti-inflammatory action and enhance inflammation resolution [138]. Inducing such polarization was evidenced for diverse BP compounds such as kaempferol [139], luteolin [140], resveratrol [141], and other phenolics [142]. Another important observation to keep in mind, but to verify by further studies, is the fact that BP appears to possess more marked effects against chronic inflammation than other bee products, which may be another advantageous argument deeming its possible use. A recent study compared the effects of BP, bee bread, honey, propolis, and royal jelly on chronic inflammation rodent models and found that BP and bee bread were more potent than other bee products [143]. From these preliminary results and considering the substantial evidence on the potential of BP against oxidative stress and inflammation, BP’s ability to promote inflammation resolution and suppress chronic inflammation settlement appears to be a promising research avenue in preventing and managing pathophysiological events in neurodegeneration and cancers.

### 3.3. Imbalances in Cell Death and Clearance

#### 3.3.1. Autophagy Modulation

Autophagy is a cellular “quality-control” process encompassing metabolic and innate-immune pathways that culminate in the degradation of hazardous and dysfunctional material present in the cytoplasm [144]. Autophagy should not be easily appraised, as it is very frequently described in aging and neurodegeneration studies, where a simplistic conception stipulates that impaired autophagy is detrimental and enhanced autophagy is beneficial for aging and neuro-performances. A complex relation between autophagy, aging and neurodegeneration pathophysiology is revealed by research, and the exact relations and interplays remain unclear [145]. Similarly, in cancers, numerous studies roughly revealed that autophagy prevents neoplastic transformation of cells and preserves their genomic integrity, but growing data reveal that the impact of autophagy in cancer pathophysiology depends on a large plethora of factors, and that autophagy may also increase neoplastic cell performances, tumor relapse, and resistance to treatments [146].

The correcting potential of BP on autophagy processes remains very poorly studied, and only a few experimental studies investigated it. A study in lipopolysaccharide-intoxicated rats recently reported that one-month feeding with BP resulted in a dramatic decrease of C/EBP homologous protein (CHOP) gene expression [147]. CHOP is known to be a key transcription factor in inducing stress-related autophagy with a particular impact on the neurodegeneration process [148]. Unfortunately, the authors used a commercial BP and did not mention its botanical origin. Accordingly, a pectic polysaccharide fraction isolated from *Rosa rugosa* BP was tested in mice submitted to a high-fat diet and was reported to promote autophagy by enhancing adenosine-monophosphate-activated protein kinase (AMPK) activity and suppressing mammalian target of rapamycin (mTOR) activity in liver tissues [149]. AMPK and mTOR were classically considered as master regulators of metabolism and autophagy [150], but recent data appear to “subvert” the classical conception that ruled during the last decade. An intricate and even possibly an inhibitory role of AMPK in autophagy was reported by many recent studies [151]. In contrast, deprivation of nurse bees from pollen leads to an increased expression of the autophagy-related (ATG) genes [152], which is a series of genes mainly orchestrating macro-autophagy but also appearing to be involved in other known forms of autophagy, namely directly in some types of micro-autophagy [153]. Many ATG genes have recently been identified as causative or risk factors of neurodegenerative, cardiovascular, and metabolic diseases, a relationship that is still by far less clear in cancer [146,153].

To sum up, the limited number of available studies proposes that BP has an intricate role in autophagy. Possible relations between numerous action mechanisms of its known components and the resulting effect of the whole product remain to be elucidated. Exact regulating mechanisms and sensors in autophagy regulation also remain not perfectly clear, a fact that unfortunately further complicates the design and conduction of preliminary experimental studies. When they were studied as pure molecules, many BP components were reported to modulate autophagy and to result in great benefits in some hurdling diseases such as NDD and cancers. However, how these molecules will behave when ingested in BP or in BP-based preparations, and how they will interact with each other when present in the same BP, remain unfortunately unknown.

Many BP compounds modulate autophagy in an intricate manner, comprising neuroprotective modulation in healthy nerve and glial cells and cell death-promoting modulation in malignant cells. Among BP compounds that have been reported to exert this duality in autophagic regulation, we cite naringenin [154], apigenin [155], luteolin [156], hesperetin [157], kaempferol [158], quercetin [159], cyanidin-3-glycoside [160], chrysin [161], pinocembrin [162], protocatechuic acid [163], ellagic acid [164], and spermidine [165]. Myriad other studies have been published on this duality and could not be reviewed here.

#### 3.3.2. Apoptosis Modulation

Contrary to what has long been thought in biomedical research, understanding and appraisal of cell death is subjected to a deep revolution in the very recent years. It has particularly been demonstrated that a clear-cut distinction cannot be made between cell death mechanisms because of their frequent overlap and mutual regulation, and the emergence of formerly unknown or poorly understood mechanisms [166,167]. Accordingly, we will see that BP acts on diverse pathways that are involved in this overlapping. The understanding of this action should therefore be with a novel holistic approach, which will certainly be advantageous in bioprospection, translational research, and disease management, either in neurodegeneration, where cell death is the main disease fatal outcome, which unfortunately remains neither understood nor stoppable, or in cancer, where resistance to cell death remains the biggest hurdle in targeting malignant cells.

B-cell lymphoma 2 (Bcl-2)-associated X protein (Bax) is the key initiator of the intrinsic (mitochondrial) pathway of apoptosis by being translocated from the cytosol to the mitochondrial outer membrane and then “spark” a quasi-non-returning point toward cell death by creating mitochondrial permeabilization pores and oligomerizing [168,169]. Targeting Bax and other key apoptosis effectors from the Bcl-2 family was proposed as a feasible approach to considerably overcome cancer cell resistance to apoptosis [170]. Bax is also activated, jointly with the caspase cascade, by the accumulation of neurotoxic misfolded proteins such as Aβ and phosphorylated Tau [171] and was generally found to be overexpressed in many major NDDs [172]. On the other hand, tumor protein 53 (p53) is a central effector in many stress-related molecular cascades by mainly acting as a tumor suppressor [173]. When muted, p53 mediates the generation of malignant cells that excessively proliferate despite their muted genome [174]. Moreover, cytochrome C generates the active form of caspase-9 which induces the effectors caspase-3 and caspase-7 that, besides cleaving hundreds of proteins, undergo a series of cell-demolishing processes to mediate both intrinsic and extrinsic pathways of apoptosis [175,176].

In programmed cell death, BP has been reported to intricately modulate apoptosis in cell lines and experimental models of neurodegeneration, cancers, and other diseases. Interestingly, this modulation was expressed by inhibiting apoptosis in healthy cells and promoting it in malignant cells, as we will see throughout some examples.

A methanolic extract of *Filipendula ulmaria* BP was found to potently induce apoptosis in a dose and exposition time-dependent way in a murine colorectal cancer cell line [177]. A peptide fraction of a hydrolysate from commercial BP originating mainly from *Mimosaceae* was reported to promote apoptosis in a human lung cancer cell line in a timely-dependent manner (apoptosis rate passed 80% 72 h after cell treatment with BP extract) [178]. Involved mechanisms were not investigated by these two studies, as the assessment of apoptotic effect was performed only optically through cell counting. In a human lung cancer cell line, a polyphenolic extract of BP was reported to promote apoptosis by upregulating Bax and caspase-3 and downregulating Bcl-2 [179].

Regarding the protective effects on healthy cells, a hydroethanolic extract of *Schisandra chinensis* BP was found to inhibit cisplatin-induced apoptosis in rodent liver and kidney tissues by downregulating gene expression of apoptosis mediators Bax, p53, caspase-3, caspase-9, and cytochrome C [180]. Accordingly, in rat models of propionic acid-induced autism, BP feeding resulted in significantly reducing caspase-3 levels in animal brains [115]. The authors used a commercial BP and did not report its botanical origin or composition. Likewise, BP feeding was found to markedly protect rat pups against prenatal methylmercury-induced neurotoxicity. Caspase-3 inhibition was among many mechanisms that this study found to be involved in this protection [181].

#### 3.3.3. Ferroptosis Modulation

Ferroptosis is a newly discovered form of programmed cell death (first described in 2012), mainly triggered by an intracellular overload of iron and a deficiency in glutathione peroxidase 4 (GPX4); the former resulting in an increased reactive species production through Fenton reactions and the latter resulting from glutathione depletion [182]. These two factors then result in an accumulation of lipid peroxides and subsequent cell death ultimately driven by membrane lipid peroxidation [183]. Other glutathione-independent inhibitors of lipid peroxidation have been discovered and may therefore act as regulators of ferroptosis [182,184]. Ferroptosis has been implicated in many aging-related alterations such as cellular senescence [185] and inflammation to which ferroptosis is bidirectionally related [186]. It has been especially found to crucially participate in neurodegeneration pathogenesis and to inhibit malignant cell proliferation, hence being coveted as a therapeutic target both through its induction in cancer cells and its inhibition in neurodegeneration [187,188].

Some mechanisms directly related to ferroptosis, such as reducing the levels of lipid peroxidation byproduct MDA (e.g., *Fagopyrum esculentum*, *Schisandra chinensis*, and other multifloral BPs) [189,190], and increasing glutathione levels (e.g., *Schisandra chinensis*), were reported for some BPs in animal models and cell cultures [11], but direct assessments of the anti-ferroptosis effect of BP as a whole product or an extract are still lacking.

However, a wide range of polyphenols, including many ubiquitous BP compounds, have especially been shown to exert potent ferroptosis modulation. Quercetin, apigenin, and gallic acid, for example, were reported to markedly reduce the viability of many cancer cell lines through ferroptosis induction while significantly preventing this cell death form in different non-malignant neuronal lines [191,192,193,194,195,196]. Numerous phenolics, including those present in BP, have been reported to exert similar effects but could not be detailed here (see good recent reviews in [197,198]).

Diverse other BP compounds have been verified to exert important ferroptosis modulatory effects. Uronic acid was reported to act as ferroptosis promoter in ovarian cancer cells [199]. This polysaccharide compound has been found at high concentrations in a *Lycium barbarum* BP extract and drove the antioxidant potential of the polysaccharide fraction of this extract [200]. Carotenoids and retinol metabolites have also been reported as ferroptosis inhibitors. Lycopene inhibited chemically induced ferroptosis in the mice hippocampus [201]. Lutein’s metabolites in the human body, viz. 3′-epilutein and 3′-oxolutein, inhibited glutamate-induced ferroptosis in a neuronal cell line [202]. β-carotene and vitamin A have been reported to inhibit ferroptosis and are both endowed with a high potential to cross the blood-brain barrier (BBB) [203,204]. Vitamin A and its metabolites inhibited experimentally induced ferroptosis in neuronal and non-neuronal cells [205].

Vitamin C was also shown to intricately intervene in ferroptosis regulation. In murine normal cells, it prevented chemically induced ferroptosis [206], while it manifested a selective ferroptosis-mediated killing of pancreatic cancerous cells [206]. Vitamin E is also an acknowledged endogenous ferroptosis inhibitor due to its effective lipid peroxidation-inhibitory potential [203,207] and this has been shown to mediate advantageous effects in some clinical and preclinical studies of NDD [208,209]. Selenium is another key element in the ferroptosis process and regulation and is abundantly present in some BPs, as we have already seen. It is a crucial component and activity determinant of selenoproteins such as GPX4 [210]. Its supplementation upregulated GPX4 expression and increased resistance to ferroptosis in cell cultures and animal models [210]. Multiple studies reported that selenium protects against ferroptosis in NDD [211]. Coenzyme Q10 was recently found to play crucial roles in preventing ferroptosis, mainly through regulating lipid peroxidation in the plasma membrane [212]. Other nutrients that are known as universal BP compounds are directly involved in regulating ferroptosis but are not discussed in this paper due to their very expanded and complex roles and interactions (e.g., unsaturated fatty acids and other lipids, amino acids such as cysteine, glucose and its sources, and minerals such as iron). They have been well reviewed in recent studies [210,212].

Zinc is perhaps the last unveiled element in ferroptosis. It has very recently been identified to be essential for ferroptosis by discovering that a zinc chelator suppressed ferroptosis, and zinc addition promoted ferroptosis, even with iron chelation [213]. Treatment of different cancer cell lines with high levels of zinc induced ferroptosis [214]. Zinc is also known to be deeply involved in central nervous system (CNS) functions and neurodegeneration. It was found to suppress ferroptosis in animal models of spinal cord injury [215]. Zinc-mediated ferroptosis modulation, either through inhibiting it in neurodegeneration or selectively inducing it in cancer cells, appears therefore to be a novel therapeutic targeting avenue.

### 3.4. Metabolic and Nutritional Disorders

Metabolic imbalances are widely viewed to instigate or accelerate carcinogenesis and neurodegeneration, especially when chronically settled in the human body. Metabolic dysfunction, roughly defined as some or all metabolic syndrome criteria combined in one person, has been implicated in the pathogenesis of at least thirteen types of cancers and is considered responsible for at least 6% of acquired cancers worldwide [216].

The importance of BP in preventing and managing diverse aspects of the deleterious interplays between nutrition, metabolic disorders, and cancer appears to be well-founded in our point of view due to the substantial amount of preclinical data that highlights BP importance in overall nutrition- and age-related diseases, as well as in their major accompanying triggers such as oxidative stress and chronic inflammation. Due to its high and relatively easy-to-adapt variability, low to moderate caloric value, richness in all necessary nutrients for humans, and potential safety, we can hypothesize that BP is a notable candidate to drive dietary interventions in cancer prevention and clinical management. It is also noteworthy to mention that numerous known bioactive components of BP (e.g., polyphenols) are known to intricately and advantageously modulate autophagy depending on the healthy or abnormal state of cells, and to tackle oxidative stress, and inflammation as we have detailed in this work and in our recent publication [11]. Moreover, since modulating autophagy, oxidative stress and inflammation are the main highlighted mechanisms to explain the benefits of caloric restriction in cancer and neurodegeneration [217,218,219], the importance of BP in such a context can be further deemed. Some calorie restriction mimetics have been actively studied recently and are being reported to produce caloric restriction effects without the need to cut caloric supply. These candidates include, for example, apigenin, quercetin, resveratrol, and spermidine [220,221,222]. Interestingly, these examples are reported to be present in many BPs and are endowed with fast-growing experimental and clinical evidence, which opens new research horizons for novel BP-based candidates. Likewise, a large body of evidence connects metabolic dysfunctions to neurodegeneration initiation and exacerbation. Caloric restrictions have been extensively reported to decelerate the aging process and to tackle neurodegeneration mechanisms in animal models, with autophagy, oxidative stress, and inflammation modulations being reported among the major involved mechanisms [217,222,223]. Given these considerations, BP appears to be a distinct candidate in dietary management of neurodegeneration, either in the early risky stages or during the disease course.

On the other hand, the possible potential of BP in preventing and holistically managing metabolic diseases was investigated by a considerable number of studies. A review of the most recent ones was completed in our recent publication, which can be consulted for more details [11]. Briefly, BP, either from monofloral or multifloral sources and from different geographical origins, was found to enhance digestive health and function and repair digestive tract injuries, exert numerous hepatoprotective activities and enhance liver functions, lower glycemia, and possess many other antidiabetic properties, correct dyslipidemia by reducing cholesterol and/or triglyceride levels, reduce body weight, and tackle numerous obesity mechanisms [11]. BP’s potential to enhance digestive microbiota and correct its dysbiosis will be discussed in the next subsection. Moreover, it is well known that at least some metabolic diseases are tightly linked to the development of neurodegeneration and cancers. Diabetes mellitus, for example, has been recently widely linked to AD, and the latter is frequently called type 3 diabetes [224]. Obesity is also directly linked to some cancer types, as we have already seen. BP may therefore also be of great interest in preventing metabolic disease comorbidities, especially those that may promote NDD or cancer pathogenesis. Owing to the amount of evidence that we expose in this publication, the use of BP in patients suffering from metabolic diseases may be advocated under specialized supervision and according to personalized data as a part of clinical programs to prevent secondary complications towards more deleterious diseases, especially in persons being at increased risk to develop neurodegeneration and cancer. However, more solid and reliable preclinical and clinical data on this specific context are needed.

### 3.5. Microbiota Alterations

Human microbiota studies have emerged at an incredible rate in the very recent years due to the discovery of very complex and diverse interferences of this micro-world with human physiology, health, and diseases. The keyword “microbiota” strikingly returns more than 97,000 scientific works published during the last five years in the PubMed database (consulted on 10 December 2024). The gut microbiome manifests a great variability and is now very well known to determine or modulate a large array of health- and disease-related aspects. This variability is linked in a great part to modulable factors, of which nutritional and lifestyle ones are the most determinant [225]. A healthy microbiota has been found to potentiate cancerous process prevention and suppression, while some infectious or pro-inflammatory strains in dysbiosis may exert many tumorigenesis- and metastasis-enhancing effects and colonize local and distant tumors [226,227]. On the other hand, gut microbiota has been shown to play pivotal roles in neurogenesis, neurodevelopment, microglial homeostasis and activation, cognitive and behavioral functions, and a large plethora of other neurological processes [228,229]. Gut dysbiosis is tightly and bidirectionally linked to a wide range of neurodegeneration triggers such as oxidative stress, neuroinflammation, aberrant protein deposition, and other structural, biochemical, and functional alterations inside the CNS [230,231].

BP is rich in a large plethora of nutrients and other bioactive compounds that have been widely reported to exert manifold enhancing effects on gut microbiota. An extensive number of studies unveiled important microbiota-modulating effects of polyphenols, especially flavonoids, with numerous ones frequently reported in BP (see recent dedicated reviews [232,233]. Carotenoids are also endowed with substantial evidence supporting their benefits on gut microbiota via different mechanisms such as promoting beneficial strains and reducing harmful ones, suppressing pro-inflammatory signaling, mitigating gut alterations, and ameliorating intestinal wall structure and functions [234,235]. In addition, other natural compounds that are present in BP, viz., phytosterols, glucosinolates, and betaines, were also repeatedly described for their ameliorating effects on microbiota in animals and humans [79,89,236,237].

Vitamins and minerals, which are present in considerable amounts in BP [11], have a great and complex impact on microbiota. Vitamin A indirectly enhances microbiota diversity and functions by promoting mucin production [238]. B vitamins are essential for gut microbiota growth and metabolism [239]. They exert diverse modulatory effects on this microbiota and its surrounding environment, while microbiota also exerts a very significant influence on vitamin B metabolism and functions [239]. Vitamin E can regulate gut microbiota directly and indirectly, while gut microbiota may have a great influence on vitamin E metabolism and fate [240,241]. Studies in humans reported that vitamin C enhanced microbiota diversity, evenness, and beneficial strains [240,242]. Microbiota is involved in the liberation from food matrix and digestive absorption of vital minerals, while many of these elements are known to enhance microbiota diversity, prevent gut dysbiosis, and/or regulate microbiota metabolism [243,244].

Carbohydrates, the most abundant BP compounds, exert diverse effects on microbiota depending on their structural differences (for recent comprehensive reviews of these effects, see [245,246]). BP may also be a rich source of dietary fibers, as we have explained. With a good fiber supply, BP can modulate gut microbiota via diverse mechanisms that have been verified by experimental studies [108,247], such as enhancing SCFA production, promoting some advantageous strains, and ameliorating microbiota diversity and the *Firmicutes*/*Proteobacteria* ratio. The latter have been considered as an indicator of gut microbiota health and a biomarker of proneness to developing many diseases such as metabolic disorders, cancers, and some NDDs, including dementia [248,249]. Clinical studies that assessed the effects of prebiotic dietary fiber-based microbiota modulation on some cancers (e.g., melanoma) and NDD (e.g., autism) reported generally that observed effects were rapid and reversible, which suggests that a long course of these interventions may be more helpful [250,251]. Due to its composition (own microbiome, prebiotic fibers, micronutrients, etc.) and safety, BP holds great potential to mimic such interventions.

In addition, BP bears a valuable microbiome comprising mainly *Lactobacillus* strains and others, such as *Pseudomonas* genus bacteria and *Saccharomyces* genus yeasts, and remaining poorly exploited despite its potential [11]. More specific roles, such as vitamin synthesis, immune system development and performance mediation, microglia enhancement, and angiogenesis modulation, are attributed to gut microbiota [252]. The multifaceted and positive effects of BP on gut microbiota may thus potentiate these roles and thus result in a beneficial impact in cancer and neurodegeneration pathophysiology.

### 3.6. Infections

Some infectious diseases or long-lasting minor or localized infections have been shown to trigger neurodegenerative and oncogenic mechanisms. On the other hand, the anti-infective potential of numerous BP compounds is largely known. However, studies of BP as a whole product for anti-infective potential are more recent. The anti-infectious potential of BP was largely reported against many bacterial strains but also against some viral and fungal pathogens. Many of these pathogens are involved in cancer and neurodegeneration, as we will see.

#### 3.6.1. Viral Infections

Several viral infections, including coronavirus, influenza, pneumonia, herpes, hepatitis C, and papillomavirus infections, have been linked to diverse NDD [253,254] and cancers [255,256], as well as to their related pathophysiological events.

Many recent studies have shown the potential of BP and many of its ubiquitous compounds, which are frequently present together at substantial levels, mainly phenolics, against the coronavirus SARS-CoV-2 [257,258,259]. Inflammation is a central mechanism by which this virus mediates its deleterious effects, including those linked to neurodegeneration and cancer [260,261]. Thus, in addition to its direct inhibitory effect on the SARS-CoV-2, BP, as an important anti-inflammatory and antioxidant cocktail, is expected to present an important potential in tackling this mediation. BP and some compounds that were isolated from it have also been found to exhibit inhibitory activity against many influenza strains such as H1N1, H3N2, and H5N1 [262,263]. Comparable activities were also reported for a wide range of ubiquitous BP compounds against herpes simplex virus, human papillomavirus, and hepatitis C viruses [264,265,266].

#### 3.6.2. Bacterial Infections

Bacteria involved in periodontal diseases, particularly *Porphyromonas gingivalis*, have been implicated in AD pathogenesis by numerous studies [267]. A one-month feeding of BP to mice reported a strong inhibition of *P. gingivalis* in oral cavities of animals [268]. Many phenolics that are frequent in BP separately showed marked inhibitory effects on these pathogens [269]. The very ubiquitous commensal bacteria *Chlamydia pneumoniae* is also linked to NDD and other disorders, including lung cancer [270,271]. Catechin, epicatechin, myricetin, quercetin, and rhamnetin were reported to effectively inhibit *Chlamydia pneumoniae* [272,273]. *Helicobacter pylori* has also been implicated in many human diseases, including gastric cancer and ND [274,275], via mechanisms involving mainly its high potential to alter and circumvent the host’s immune response, thus culminating in prolonged inflammation, redox imbalance, and epigenetic alterations [274,276]. Based on the already elucidated unequaled potential of BP in fighting chronic low-grade inflammation and the fact that some BP compounds have been reported [277,278] for their marked inhibitory effects against this germ, we can firmly advocate preclinical and clinical research works, which unfortunately remain absent on BP as an apart product in this topic.

#### 3.6.3. Fungal Infections

The commensal fungi *Candida albicans* has also been linked to cancer [279] and neurodegeneration [280]. Many BP samples showed a potent activity against *C. albicans* and other bacterial strains, with the activity extent not always correlating with total phenolic contents [247]. Studies of other multifloral or botanically unidentified BP samples reported that the sensitivity of *C. albicans* and other *Candida* species to these samples ranged from very high to absent depending on the study [247,281,282].

### 3.7. Genetic and Epigenetic Alterations

Genetic and epigenetic alterations are among the most noticeable other mechanisms that are implicated in the pathophysiology of both neurodegeneration and oncogenesis. A wide range of intra- and extra-cellular factors can induce deoxyribonucleic acid (DNA) damages and ribonucleic acid (RNA) defects, which are known to have critical roles in numerous NDDs [283] and cancers [284].

#### 3.7.1. DNA Damage

DNA damage response (DDR) is a vital process that declines with age, but other factors can compromise it and therefore result in unrepaired or mistakenly repaired DNA damage [285]. The latter is a well-established contributor to aging by inducing cell death and senescence but has also been recently verified to induce inflammation, implicating a newly unveiled role in inflammation, which is a major culprit in aging and age-related disease [286]. Insufficiency and chronic activation of DDR may result in sustained neuronal dysfunction and consequent death [287]. DNA damages interact also with neuronal plasticity in a mutual triggering manner, resulting in a vicious cycle of altered neurotransmission and impaired DDR, and culminating in cognitive decline [285]. Defects in DDR are also a pivotal phenomenon in triggering the carcinogenic process. They fuel tumorigenesis through excessive genomic instability but also make malignant cells more prone to further alterations of DNA and in host immune response against oncogenic processes [288].

An ethanolic extract of *Castanea sativa* BP drastically reduced DNA damage byproducts by 34% in vitro [289]. Protective roles against experimental DNA damages were also evidenced for aqueous and ethanolic extracts of *Actinidia arguta* BP [290] and an ethanolic extract of a multifloral BP [291]. Polyphenols in general enjoy strong preclinical evidence as agents that lower DNA and other cellular damages and thus manifest an important potential in fighting related diseases, such as cancer and NDD [292]. Carotenoids are also ubiquitous compounds in BP that may encompass preventive and corrective effects against DNA damage [293,294]. Carotenoid effects against DNA damage may obviously not emanate only from their known antioxidant potential, but further studies are needed to elucidate this eventuality. Many vitamins and minerals have also been shown to prevent DNA damage in different contexts but could not be detailed here.

##### Epigenetic Regulation

Epigenetic regulation, which is mediated by three major types of mechanisms, i.e., DNA methylation, histone modification, and non-coding RNA, is a major genome modulator that may shape human phenotype and thus deeply contribute to defining health and disease factors and critically mediate numerous pathological events, including those implicated in cancer and neurodegeneration [295,296]. Although playing a key role in genetic expression and being inheritable and transmissible during cell division, epigenetic modifications can be reversed and are fortunately ”reprogrammable” or “erasable” due to pharmacological and nutritional interventions [295,296]. In this context, BP, as an unequaled nutritive resource and a rich pool of bioactive compounds, may represent a potential tool to carry out such interventions.

Many BP-ubiquitous phytochemicals have been found to modulate major epigenetic mechanisms. Some of them potently suppressed oncogenic epigenetic signaling and promoted epigenetic induction of tumor suppressor gene expression in experimental studies. Polyphenols are widely reported for their countless effects resulting from epigenetic modulatory mechanisms in neurodegeneration and cancer pathophysiology (good recent reviews can be found in [297,298]). All these effects are not limited to differentiated, fully functional cells. The three major mechanisms of epigenetic modulations have also been verified for numerous polyphenols in cancer stem cells, which play a crucial role in cancer renewal and resistance (reviewed in [299]).

## 4. BP and Neurodegeneration

Despite the great impediments in understanding the pathophysiology of NDD, many common mechanisms are widely recognized and known to be always involved in the disease course. Oxidative stress [300], neuro- [301] and systemic [302] inflammation, and metabolic disorders, especially metabolic syndrome and diabetes [303], are today among the well-known interfering triggers in the long course of neurodegeneration settlement. Other pathophysiological mechanisms that remain less understood, and for which targeting did not greatly succeed in clinical applications, are more specific to each disease. This includes, for example, acetylcholinesterase activity, which is clinically used in AD pharmacotherapy with a limited efficacy rate, and monoamine oxidases circulating levels, which are also used as targets in Parkinson’s disease (PD) treatments with a limited clinical success rate. All these examples of pathophysiological mechanisms have been targeted by BP, and encouraging results were reported, but studies remain very scarce and all in the very early stages of screening and prospection, as we will see.

In such context and given that multitargeting is becoming a universally coveted approach in preclinical and translational research regarding NDD, BP evidently arises as a “perfect” candidate owing its importance to its very diversified and balanced composition. In the very early triggering of neurodegenerative processes, BP may virtually be of great importance in fighting oxidative stress, malnutrition, metabolic disorders, and chronic low-grade inflammation. Its widely recognized potential for these indications is clearly valuable, especially in such initial stages. Other pathophysiological mechanisms are widely verified to be linked to neurodegeneration but remain less studied than the previous one. They include some that have been recently deeply investigated, such as ferroptosis and epigenetic mechanisms, and others that are less understood and more controversially implicated in NDD, such as autophagy- and microbiota-related effects. All these mechanisms are modulated by BP or some of its compounds in complex and promising ways, as we have long discussed. In addition to these mechanisms, which are, at a certain level, shared between neurodegeneration and cancer, BP showed interesting potential in other neurodegeneration-specific processes such as misfolded protein aggregation and glial cell-mediated neuroinflammation.

Some major translational issues remain to be solved with BP in the case of any prospective use in tackling NDD. The first one is the insufficient understanding of BP metabolism in the digestive tract and especially the modifications mediated by gut microbiota, which may even imply the microbiota-driven de novo synthesis of some compounds that are already present in BP, as we have seen for some examples. In other cases, these modifications may consist of very complex interactions involving gut microbiota but also other localizations and physiological microenvironments, as is the case in the metabolism of BP phenolic compounds. The second challenge is the insufficient understanding of pharmacodynamic and pharmacokinetic behavior and fate of all BP compounds at the systemic level as well as inside the CNS. Third, the ability of BP compounds to pass the blood-brain and blood-cerebrospinal fluid barriers and the effects that these compounds may exert in these barriers remain largely unknown. Fourth, many translational challenges originate from the persistent shortfalls in standardizing BP research and the difficulties in ensuring the reproducibility of its investigated compositions. The fifth major translational challenge emanates obviously from the poor understanding of neurodegeneration pathophysiology, which will impact any preventive or therapeutic intervention.

### 4.1. BP in Neuroinflammation

Anti-inflammatory properties of BP have been described in the CNS by experimental studies. Feeding of rodent models of autism spectrum disorder with BP remarkably decreased pro-inflammatory cytokines and mediators such as IFN-γ, IL-1α, IL-6, TNF-α, and VEGF and increased the anti-inflammatory cytokine IL-10 in animal brains [115,304]. This effect was accompanied by a significant decrease in caspase-3 and oxidative stress markers, in addition to the correction of neurotransmitter defects. Unfortunately, neither geographical nor botanical origins of used BP were reported by the authors.

Stress, especially in its chronic conditions, is known to be a potential risk factor for neurodegeneration through causing a series of neurobiological alterations, including the induction and maintenance of neuroinflammation [305]. In rats with chronic immobilization stress, BP was found to suppress neuroinflammation (mainly evaluated by TNF-α and IL-1β levels in the hippocampus) and concomitant oxidative events and to enhance corrective mechanisms such as brain-derived neurotrophic factor (BDNF) levels and antioxidant defense, in addition to the reduction of the anxiety-like behavior [306].

In the same context, another study in rodents reported that BP presented marked protective properties against neuroinflammation (evaluated mainly by IFN-γ levels) and related mechanisms (e.g., neural cell apoptosis and neurotransmitter defects) in neonates delivered by mothers that were treated with methylmercury before [181]. Another newer study reported comparable results [307] viz. neuroinflammation and oxidative stress inhibition, excitotoxicity reduction, and neurotransmission disturbance correction in rat pups, but unfortunately used a mixture of BP and probiotics and did not evaluate the effects of BP alone to draw comparative inferences.

These preliminary data, added to those available from numerous studies that evaluated the anti-inflammatory effect of BP in different anatomical locations and at systemic levels (see our previous publication [11] for a comprehensive review), as well as the multiple enhancing effects of BP on gut microbiota that we have detailed previously in the current work, unveil a very interesting potential of BP to mitigate neuroinflammation etiologies and mechanisms. The essence of focus and effort should, for now, go to the roles of different components and the very likely presence of synergistic mechanisms, as well as solving pharmacokinetic and bioavailability issues, which remain among the most difficult challenges in exploiting the great nutritional and pharmacological potential of BP.

### 4.2. BP Enzyme Inhibitory Potential

Concerning the enzyme inhibitory potential, a limited number of studies have been conducted until now on BP, mainly against known enzymes that are implicated in AD pathogenesis. Despite the encouraging results from preliminary studies, as we will see hereinafter, investigations are still in embryonic stages, and many aspects remain to be studied even in in vitro phases, such as the role of each of the BP compounds and the possible synergies that appear most likely to exist between many ubiquitous BP components.

An in vitro study of 18 different BP samples recently reported that their hydroethanolic extracts downregulated betasecretase-1 (BACE1, the cleaving enzyme of amyloid precursor protein (APP) that generates amyloid beta (Aβ) protein) activity, with multifloral pollens being more potent than monofloral ones [52]. This study, which is to our knowledge the first published one about this bioactivity, reported also that the 18 pollens did not show a strong inhibition of acetylcholinesterase (synaptic acetylcholine cleaver).

A recent in vitro study of *Sabal blackburniana* reported that the methanolic extract of pollen grains from this plant manifested an important inhibitory activity of the acetylcholinesterase [308]. Interestingly, this study noted that the IC_50_ of the anti-acetylcholinesterase activity of pollen extract (0.5 µg/mL) was dramatically reduced by the encapsulation of these extracts in zinc oxide nanoparticles (it became 0.064 µg/mL). Moreover, the IC_50_ of BP extract was even slightly lower than that of donepezil, which is a reference in the acetylcholinesterase inhibitory treatment for AD. Unfortunately, this study did not investigate the BP of the same plant to figure out a possible difference between the pollen grains collected manually from the plant and those crafted by honeybees. An older study of diverse BP methanolic extracts [309] also reported that a multifloral pollen originating mainly from *Myrcia* spp. (60%) and *Cocos nucifera* (33.3%) presented a very potent inhibitory activity on acetylcholinesterase (IC_50_ of 3.93 ± 0.64 µg/mL), followed by samples from *Eucalyptus* spp. monofloral BP, which were also very potent (IC_50_ of 26.85 ± 1.61 and 38.38 ± 2.47 µg/mL for two different samples). Other samples from *Spondias* spp. and *Miconia* spp. also presented potent activity (IC_50_ of 71.52 ± 3.58 89.66 ± 8.28 µg/mL, respectively).

A very recent study investigated, for the first time to our knowledge, the effect of BP on catechol-*O*-methyltransferase, a key enzyme in PD pathogenesis. This study found that hydroxycinnamoyl acid amides were the major drivers of the inhibitory effect on this enzyme that was strongly exerted by some of the studied extracts [310]. Some of these molecules were largely more active than some known phenolics such as caffeic acid and p-coumaric acid. Note that some widely studied flavonoids that are repeatedly isolated in BP were reported as more potent inhibitors of catechol-*O*-methyltransferase (e.g., IC_50_ was 0.96μM for myricetin and 1.38 μM for quercetin [311]).

### 4.3. BP Compounds and Protein Aggregation

A growing number of preclinical and clinical studies are recently focusing on tackling or delaying aging-related processes. Indeed, altered proteostasis is acknowledged as a major hallmark of NDD and of the overall aging process [312]. Diverse, widespread BP compounds have been reported to inhibit misfolded protein aggregation in the CNS. We will only review some BP bioactive compounds that were reported to impact the aggregation of most pathogenic proteins involved in the major NDDs, i.e., Aβ and tau in AD and α-synuclein in PD. Studies of BP as a whole product or extract are still absent.

#### 4.3.1. BP Compounds and Amyloid-β

Under physiological conditions in humans, Aβ isoforms consist mainly of Aβ_1-40_ (about 90% of total Aβ) and Aβ_1-42_ (about 5 to 10% of total Aβ) [313]. Aβ_1-42_ is more neurotoxic, so a higher ratio of Aβ_1-42_/Aβ_1-40_ correlates with faster aggregation and pattern alteration of spontaneously formed oligomeric species [314]. The transition from monomeric Aβ to a sufficient ratio of Aβ_1-42_/Aβ_1-40_ for oligomers and β-sheets forming is thought to happen usually 10 to 15 years before the clinically confirmed pathological state of AD [315]. The importance of natural products with known safety profiles is therefore of vital importance in early long-term mitigation of Aβ aggregation.

Numerous BP-ubiquitous flavonoids (e.g., naringenin, naringin, apigenin, luteolin, hesperidin, hesperetin, kaempferol, quercetin, and myricetin) and phenolic acids (e.g., gallic, ellagic, ferulic, and rosmarinic acids) are widely recognized for their multifaceted potential to counter Aβ genesis, aggregation, and toxicity [316,317,318]. Many non-phenolic BP compounds have also been reported to mitigate aberrant Aβ genesis, deposition, and/or toxicity, e.g.,: β-carotene [319], lycopene [320], spermidine [68], and betaine [321]. Diverse polysaccharides from plants were shown to exert anti-amyloidogenic, Aβ disaggregating, and cognition-promoting effects [322,323]. However, studies of such potential in BP polysaccharides are still lacking and highly recommended.

#### 4.3.2. BP Compounds and Tau Protein

The microtubule-associated protein tau is mainly known for its role in stabilizing and protecting neuronal cytoskeleton microtubules, as well as in regulating microtubule dynamics and axonal transport [324]. A basal level of phosphorylation, which is intricately regulated by the interplay of kinases and phosphatases, is necessary for tau physiological functions, but an alteration of this equilibrium may result in a hyperphosphorylation of tau leading to its excessive dissociation from microtubules and aggregation in the form of neurofibrillary tangles, and ultimately to neuronal death [324,325]. Tau phosphorylation is mediated by tau kinases such as glycogen synthase kinase 3β (GSK3β), cyclin-dependent kinase 5 (CDK5), and others, while its hyperphosphorylation may result from these kinases’ overexpression or from an inactivation of tau phosphatases [324,325].

Numerous BP compounds have been shown to inhibit tau aggregation. A wide range of micronutrients that are present in BP, especially many vitamins and minerals, have been linked to preserving tau homeostasis and preventing its pathology [326,327]. BP flavonoids (e.g., apigenin, quercetin, myricetin, genistein, naringenin, luteolin) and other phenolics (e.g., rosmarinic, caffeic, and ferulic acids, resveratrol) have been reported to tackle tau aggregation and pathology through diverse mechanisms, including GSK3β and CDK5 modulation and tau-mediated cytotoxicity mitigation.

#### 4.3.3. BP Compounds and α-Synuclein

Alpha-synuclein (αS) is highly expressed in diverse organelles of CNS neurons, including dopaminergic neurons of the substantia nigra pars compacta and other excitatory and inhibitory neurons [328]. αS is present in neuronal presynaptic terminals, possibly traducing a role in vesicular trafficking and synaptic transmission and plasticity, in addition to a wide range of functions that are still not fully understood [329,330]. Diverse factors, such as mutations and aberrances in post-translational modifications, proteostasis defects, and other factors, may intervene to trigger and promote αS misfolding and aggregation.

Many polyphenols, including some flavonoids and phenolic acids that are present in BP, have been shown to prevent or reduce αS aggregation. Among unveiled mechanisms, the formation of non-toxic irregular aggregates, rearrangement of preformed toxic intermediates, or disassembly of mature fibrils, in addition to microbiota-mediated modulation, were mainly reported [331,332]. Being more recent and still less coveted than in other protein aggregation pathologies such as Aβ, this bioprospection is still mainly focusing on phenolic compounds, with many of them being widely present in BP.

Apart from phenolics, other BP compounds remain poorly understood in αS-related pathology. A recent study found that retinoic acid, one of the main metabolites of BP carotenoids as we have seen, prevented the formation of intracellular αS inclusions and prevented αS-induced toxicity and dopaminergic neuronal loss in αS-injured neuronal cell lines and mice models [333]. Other studies have reported a certain interest in vitamins such as A and E and other micronutrients such as selenium in mitigating αS aggregation and pathology [334,335].

**Figure 3:**
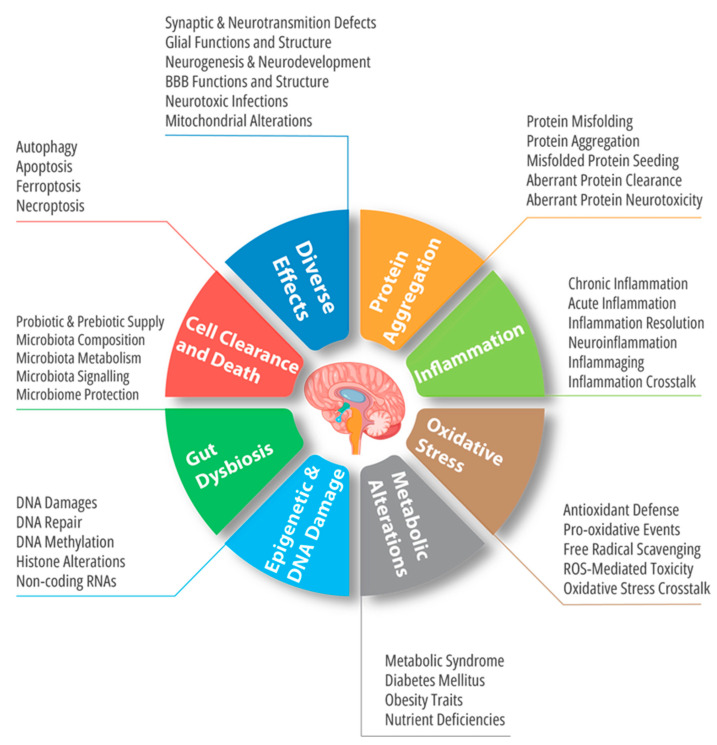
General Aspects of the Potential of BP and Its Major Compounds in Neurodegeneration.

All the potential effects of BP and its major compounds that were discussed in this section are resumed in Figure 3.

## 5. BP and Cancer

Carcinogenesis is a normal process occurring in the human organism, and the immune system should normally eradicate produced cancerous cells, but an overly complex interplay of different intrinsic, extrinsic, and behavioral factors can sustain the carcinogenic process and promote tumor formation and metastasis. Among these factors, bad dietary habits, long-lasting oxidative and inflammatory conditions, obesity, dysbiosis, epigenetic changes, some infectious diseases, and sedentary lifestyle are widely known [336,337]. Apparently, these examples of factors are all modifiable early before disease onset.

Bee products, especially BP, may be of foremost importance in such conditions. It is repeatedly reported to encompass anticancer properties by scientific experiments and reviews, and furthermore to interestingly potentiate cancer pharmacotherapies and alleviate their side effects. Main known mechanisms of BP anticancer effects include apoptosis stimulation in malignant cells, antiproliferative bioactivities, and reduction of tumor growth [338], evidently in addition to the anti-inflammatory and antioxidant effects, the possible modulation of some pre-carcinogenic troubles such as autophagic and epigenetic aberrances, and the tackling of healthy cell death mechanisms as we have discussed.

Due to its safety and rich composition, BP could be used in the long term as a prophylactic product, especially in at-risk persons. Its verified pharmacological properties could be of great help in early stages of tumorigenesis, especially if innovative formulations assemble well-targeted galenic forms and standardized compositions at efficient levels of desired compounds. The fact that other bee products have been repeatedly reported to have higher cytotoxic activities than BP, which, however, remains less investigated in cancers, should not be taken as a rule. Some studies have challenged such allegations by showing that these activities depend on the extraction process and yield, bee species, and tested cell lines. In addition, some BP extracts were more potent than other bee products [339] or than chemotherapeutic drugs in vitro on selected cell lines [340].

In addition to the extraction process, pollen preliminary preparation, treatments, and formulation will evidently also be key determinants of its activity. A protease-mediated pretreatment of BP resulted in potently active peptide fractions against a lung cancer cell line [178]. Hydrostatic treatment of BP also resulted in markedly ameliorated its active compound release, and permitted a marked increase of both its antiproliferative and anti-apoptotic activities as well as a potentiation of cancerous cell cycle destruction [341]. Fermentation was also frequently reported to enhance BP compound content, bioavailability, and bioactivities [11].

Furthermore, anticancer properties seem to be present in BP regardless of the bee species that forage it. We will see many examples, especially with BP collected by different genera and species of stingless bees, in the subsection hereinafter. Due to the scarcity of studies on BP as a whole product, we will also briefly discuss some selected ubiquitous BP compounds that we are giving as examples throughout this work.

### 5.1. Effects on Carcinogenesis and Cancerous Cells

*Nelumbo nucifera* BP was reported to exert a multifaceted effect against prostate cancer, including antiproliferative, apoptosis-promoting, and cell cycle-disturbing effects on the human PC-3 cell line [341]. Interestingly, this study reported that all treated pollen extracts (methanolic, which was the most potent, ethanolic, and aqueous) increased glutathione depletion (despite its antioxidant role, glutathione is a key element for tumor growth). The observed effect in all extracts were dose- and time-dependent.

A study of methanolic extracts of six monofloral BP samples, collected by *Apis mellifera*, on three cell lines of human breast cancer showed that some of these samples exerted an anticancer effect [342]. *Bidens pilosa* pollen had a very potent antiproliferative effect (cell viability was drastically 0% on BT-20 and Hs 578T cells and 43.5% on MCF-7 cells), while *Camellia sinensis* pollen extract had a lower but also marked effect on only two cell lines (BT-20 and Hs 578T). These two BP were the richest in flavonoids and carotenoids. *Brassica napus* and *Nelumbo nucifera* pollens did not show marked effects on the studied cell lines.

Another study of a BP originating predominantly from *Filipendula ulmaria* reported that a methanolic extract of this BP exhibited a marked dose- and time-dependent antiproliferative effect and a weak apoptosis-inducing effect on a colon carcinoma cell line [177]. Two samples were used in this study; the first contained 70% and the second 80% of *F. ulmaria* pollen. Other old studies have reported that BP had an inhibitory effect on bone marrow cancer and leukemia cell lines [343].

An ethanolic extract of BP manifested an antiproliferative activity on lung adenocarcinoma (A549) and breast cancer (MCF-7) cell lines [179]. Analysis of this extract found that it was composed of flavonoids and phenolic acids that are frequently reported in BP (naringenin, quercetin-diglycoside, catechin, and caffeic, chlorogenic, cinnamic, coumaric, ferulic, gallic, and syringic acids). Another BP ethanolic extract that was particularly rich in apigenin and benzoic acid showed an antiproliferative effect in a mouse myeloma cell line [344]. Although a tested propolis extract in this study had a higher phenolic content and antioxidant potential, BP was more active against myeloma cells than propolis.

A BP extract prepared with a hexane and methanol solvent mixture showed important anticancer properties in experimentally induced mice breast cancer [345]. In these animals, BP significantly reduced tumor size and body weight loss and increased survival rate. Interestingly, BP extract at 200 mg/kg was able to ameliorate survival rate significantly more than doxorubicin alone and to correct body weight loss while doxorubicin alone induced a greater weight loss than the one observed in untreated cancerous mice. In addition, BP ameliorated oxidative and inflammatory markers and diverse hormonal level alterations in fed animals. This study, which used a commercially botanically- and chemically-unidentified BP, reported a synergistic effect that we will discuss later.

An in vitro study used BP hydrolysates obtained by commercial proteases on a human bronchogenic carcinoma cell line and showed that some of these hydrolysates manifested a very marked apoptosis-inducing activity (up to 80% after 72 h by an alcalase-resulting fraction) [178]. Moreover, this activity was selectively exerted by BP on the apoptosis process, either in its early or late stages, and no significant effect on cell necrosis was noted. The authors used commercial pollen and did not describe its botanical origin.

Evidently, not all studies on the anticancer potential of BP reported similar results. A study of the effect of nineteen ethanolic extract samples, including mono- and multi-floral BPs, on five human cell lines of prostate (PC-3), breast (MCF-7), stomach (AGS), and lung (A549 and NCI-H727) carcinomas found that all extracts presented, at variable levels, an antiproliferative activity that was, however, much weaker than cisplatin (assessed by the inhibitory concentration IC_50_) [52]. The only exceptions were that two monofloral BP samples (*Actinidia arguta* and *Quercus palustris*) did not show a significant antiproliferative potential (IC_50_ > 25 mg/mL) in the A549 cell line.

Inhibitory activity of multiple BP samples was also tested in vitro on cell growth of gastric, colorectal, cervical, breast, and non-small-cell lung cancer cell lines [44]. In this study, most of the studied BP samples failed to show antiproliferative activity on the studied cell lines. Only one sample (originating at 100% of *Coriandrum* and *Daucus* sp.) showed significant inhibitory activity on breast cancer cells, and another sample (composed of *Olea europaea* at ˃85%) showed significant inhibitory activity on cervical cancer cells.

One study that went “against the wave” in anticancer activity of BP was recently published. This study reported that BP feeding to zebrafish models of melanoma not only did not result in a protective effect against tumor cell proliferation but promoted tumor growth in fed animals, and, although it significantly modified animal intestinal microbiota, gene transcript levels of proinflammatory mediators in the intestine did not differ between pollen-fed and control animals [346]. This brings conflictual results with some previous experimental results [338] that reported a BP activity against melanoma. We have already seen numerous examples where BP presents antineoplastic potential. In addition, many studies reported that BP may have an important tyrosinase inhibitor potential [49,104]. Studies on BP in melanoma remain extremely scarce, and such result discrepancies are perfectly normal in the absence of sufficient study sampling and diversity.

BPs from bee species other than *Apis mellifera* have also been shown to be endowed with interesting anticancer effects. A recent study reported that ethanolic extracts from BPs collected by seven stingless bees showed, at different extents with few exceptions, inhibitory effects against colorectal (Caco-2), cervical (HeLa), and breast (MCF-7) cancer cell lines [340]. BP collected by *Homotrigona fimbriata* showed a potent antiproliferative effect, which largely exceeded that of 5-fluorouracil at the same mass dosage (500 µg/mL), against all tested cancer cell lines. Accordingly, a methanolic extract of BP collected by the stingless bee *Lepidotrigona terminate* showed a significant dose-dependent antiproliferative effect on a breast cancer cell line (the IC_50_ was 15 mg/mL on MCF-7 cells) [347]. The authors, however, did not mention the botanical origin nor the approximate composition of BP, but they reported that it presented a marked antioxidant activity.

A recent study that used a pollen, collected by the stingless bees *Trigona* spp. without indicating its botanical origin, reported that water extract of this pollen had a certain antiproliferative activity on a human breast cancer cell line (the IC_50_ value was 18.6 mg/mL on MCF-7 cells), and that activity was similar to that of water extract of propolis [348]. Interestingly, this study reported that measured antioxidant potential was greater in BP water extract than in propolis or honey water extract (honey extract did not show a significant effect on the studied cell line). Another interesting observation reported by this study was the fact that pollen water extract was less toxic than propolis water extract on a normal human keratinocyte cell line. This study, being performed only in vitro, evidently needs further investigations. The reported observations about selective toxicity on cancerous cells and the higher potential and safety of pollen, as we have already seen also in a prostate cancer example, commend a potential use of BP in anticancer bioprospection. All given examples, and generally all available data until nowadays, provide an encouraging and considerable amount of experimental evidence, but which remains still in the “embryonic” stage clinically.

A study of BP collected by three stingless bee species reported that all three types of pollen manifested antiproliferative activity on human breast adenocarcinoma cell lines, although obviously at different extents [349]. However, BP collected by *Geniotrigona thoracica* presented markedly higher potential than that collected by *Heterotrigona itama* and *Tetrigona apicalis*. A similar selective effect on cancerous cells as the one reported by the previous example [348] was also reported by this study. *Geniotrigona thoracica* BP exerted marked antiproliferative activity and presented a much higher therapeutic index when assessed by reference to a human mammary epithelial cell line taken as control normal cells [349], which indicates that this pollen exerted a selective antiproliferative effect on cancerous cells with higher safety regarding normal cells.

### 5.2. Effects on Anticancer Therapies

In addition to its direct anticancer potential, BP has been reported to exert multiple advantageous effects on other anticancer therapies. Specifically, BP was able to reduce the toxicity of chemotherapeutic agents and enhance the effectiveness of anticancer chemo-, radio-, and immunotherapies in a multitude of in vitro and animal models of cancers. A multitude of anticancer mechanisms were unveiled by these studies and included the suppression of multidrug resistance effectors, the promotion of apoptosis and cell cycle arrest induction, the stimulation of mitochondrial altering effects, and diverse other molecular mechanisms. We will briefly see the studies of BP as a whole product or extract and give some examples of the most abundant bioactive compounds of BP.

#### 5.2.1. Reducing the Toxicity of Anticancer Therapies

A water extract of BP markedly reduced cisplatin-induced histological alteration in hepatic, renal, and testicular tissues of mouse models [350]. Moreover, this extract prevented the genotoxic and cytotoxic effects of cisplatin in bone marrow cells, in addition to correcting the aberrances in multiple oxidative stress indicators in intoxicated animals. It is noteworthy that the preventive effect of BP was more pronounced than that of propolis extracted with a similar protocol [350]. BP botanical origin and chemical composition were not investigated in this study. Accordingly, BP from *Schisandra chinensis* prevented many cisplatin-induced toxic alterations in the livers and kidneys of rat models [180]. The observed preventive effects consisted mainly of reducing tissue degeneration and correcting the levels of diverse inflammatory, oxidative stress, and apoptosis mediators and regulators, in addition to markedly reducing the alterations in hepatic transaminases, blood urea nitrogen, and creatinine levels. Water extract of another commercial BP was reported to counteract the doxorubicin-induced suppression of hematopoiesis in rat models [351]. BP feeding reduced bone marrow and spleen histological alterations and increased spleen and body weight and survival rate, which were all altered by doxorubicin, in addition to preventing the decline in the abundance of diverse blood cells, including red and white blood cells and platelets. At the molecular level, BP corrected many alterations in hematopoiesis inducers, inflammatory mediators, and oxidative stress and apoptosis markers. Used BP showed a high safety profile in the animals studied. Another in vivo study reported that BP feeding protected rats against methotrexate-induced testicular damage [352]. Water extract of BP completely corrected the decrease in testosterone and the increase in luteinizing hormone and significantly suppressed the alterations in oxidative stress markers, testicular and epididymal weights, and sperm structure and composition that were all induced by methotrexate injection. The authors used a commercial BP and did not indicate its geographical and botanical origins or chemical composition.

Some major BP compounds have also been reported repeatedly to prevent chemotherapeutic-induced toxicity. All the examples that we will report hereinafter have been realized in murine models, and the underlying mechanisms appeared to rely mainly on the antioxidant, anti-inflammatory, anti-genotoxic, and anti-apoptotic potential of the studied compounds. Evidently, most reported studies concerned flavonoids. Investigated organs where such protective effects were observed were mainly the liver, kidney, spleen, heart, lung, and nervous system. Polyphenols, mainly flavonoids and phenolic acids, including many of those ubiquitously present in BP, have been extensively studied for their chemoprotective potential in vitro and in vivo. Some examples that showed those protective effects in vivo were naringenin [353], luteolin [354], hesperidin [355], quercetin [356], apigenin [357], kaempferol [358], isorhamnetin [359], myricetin [360], pinocembrin [361], and protocatechuic [362], ellagic [363], chlorogenic [364], rosmarinic [365], and ferulic [366] acids. Other BP compounds, such as lutein [367], lycopene [368], choline [369], and coenzyme Q10 [370], have also been reported to mitigate the toxicity of anticancer therapies.

It is also important to note that many of the studies that investigated the combination of these natural compounds with chemotherapeutic agents reported a synergistic potential and an achievement of the desired anticancer effect with a lower drug dosage. This may evidently imply that reducing the necessary dose will also be a potential way to reduce chemotherapeutic toxicity to non-malignant cells and to preserve diverse physiological functions. Moreover, combinations of these BP compounds together generally resulted in a greater chemoprotective effect. This was also the same for combining these natural bioactive compounds with known protective drugs that are already used in clinical practice. Indeed, a combination of naringenin with metformin resulted in a synergistic potential to markedly reduce the necessary dose of liposomal doxorubicin to achieve a similar therapeutic effect in xenograft models of breast cancer [371]. Quercetin and resveratrol combination markedly reduced doxorubicin cardiotoxicity, which is the major impediment of this important antineoplastic agent [372]. Myricetin and quercetin also had a synergistic preventive effect against cisplatin-induced nephrotoxicity in mice [373]. Histopathological alterations were not completely prevented by either of the two flavonols, but their combination almost normalized the histological aspect of the microscopic slices. Other studies with similar results are available in literature. These synergisms roughly suggest the important potential of more combinations of chemoprotective bioactive compounds in cancer management. Such combinations are unequally abundant in BP from all botanical and geographical sources, but the real extent of eventual synergisms remains to be unveiled by further studies.

In addition to chemoprotection, some BP compounds have also begun to be recently reported to reduce the toxicity of cancer immunotherapies. In murine models, chrysin markedly prevented trastuzumab-induced cardiotoxicity [374], while resveratrol prevented pembrolizumab-induced hepatotoxicity and neurotoxicity [375]. Coenzyme Q10 mitigated trastuzumab toxicity in human cardiomyocyte and hepatocyte cultures [376]. This kind of protective effect is of great importance if sufficiently verified with other BP compounds and studied for their possible synergisms. Immunotherapies have revolutionized cancer management, and their side effects appear to emanate mainly from their ability to modulate immune processes, while their combination with chemotherapies, which may hold greater promises in some cancers, is hindered by cumulative toxicities [377].

Additionally, many BP compounds have shown important radioprotective effects in experimental studies. Evidently, phenolic compounds were the most studied, but other BP bioactive compounds and micronutrients have also been shown to encompass important protective potential against radiotherapy injuries. This kind of protective effect was especially reported for flavonoids (e.g., anthocyanins, apigenin, genistein, quercetin, and naringin) and phenolic acids (e.g., caffeic, chlorogenic, cinnamic, ferulic, gallic, and rosmarinic acids) [378,379,380]. Radioprotective effects were also evidenced for carotenoids, including β-carotene, lutein, zeaxanthin, and lycopene; coenzyme Q10; vitamins A, B, C, and E; zinc, and selenium [380,381,382].

#### 5.2.2. Enhancing the Effects of Anticancer Therapies

An ethanolic extract of *Trifolium alexandrinum* BP was found to synergistically enhance bevacizumab activity against A549 lung cancer cell lines and reduced its IC_50_ [383]. Similar synergistic potential has also been reported on MCF-7 breast cancer cells, where BP combination minimized the required dose of bevacizumab to achieve a similar anticancer effect [179]. Ethanolic extract of *Trifolium alexandrinum* BP was additionally verified to synergistically potentiate bevacizumab anticancer effects, including the reduction of tumoral nodule incidence and size and the activation of diverse proapoptotic and other molecular anticancer mechanisms in murine models of non-small lung cancer [384]. Methanolic extract of a BP collected by the stingless bee *Lepidotrigona terminate* potently enhanced the antiproliferative effect of cisplatin even when the latter was used at low doses on a breast cancer (MCF-7) cell line. A dose of 15 mg/mL of BP quasi-doubled the antiproliferative effect of cisplatin even at its lowest used dosage (2.5 µmol/L). BP was also reported to synergistically promote the cytotoxicity of 5-fluorouracil and bee products on the HTC-116 human colon cancer cell line [385]. In this study the IC50 of 5-fluorouracil was reduced from 6.94 to 1.90 µM when combined with BP. Interestingly, the potentiating effect of BP was more potent than that of honey or royal jelly.

BP also acted synergistically with immunotherapies. A nano-encapsulated ethanolic extract of BP synergistically exerted an antiproliferative effect on a non-small lung cancer cell line in combination with Bevacizumab [179]. Another BP extract was found to significantly enhance apoptosis induction and tumor volume and growth reduction that were exerted by Bevacizumab in mouse models of chemically induced lung cancer [384]. Finally, a small clinical trial reported that BP ameliorated menopausal symptoms in breast cancer patients receiving antihormonal treatment [386].

Many BP-ubiquitous compounds were reported to enhance chemotherapeutic effects on cancer cells. In an aim to overcome the two major challenges of cancer management, viz. toxicity and resistance, the combination of small molecules, especially polyphenols, with traditional anticancer therapies has been extensively investigated recently. Numerous studies have shown that phenolics (including many of those found in BP) suppress multidrug resistance and synergistically enhance anticancer effects of chemotherapies. These compounds encompass many pharmacokinetic amelioration capabilities toward chemotherapeutic drugs, such as reducing drug efflux, which is the main cause of multi-drug resistance in cancer cells, enhancing drug cellular uptake, and modulating drug metabolism. All these results were thoroughly reviewed in recent studies [387,388,389].

In addition to enhancing the effect of chemotherapeutic agents on cancer cells, many bioactive compounds that are present in BP have been shown to sensitize malignant cells to radiotherapy. This was, for example, evidenced for many phenolics such as quercetin, kaempferol, genistein, ellagic acid, and resveratrol [390].

### 5.3. Anticancer Potential of BP Compounds

A wide range of BP compounds have been highlighted for their important cytotoxic, antiproliferative, antimetastatic, antiangiogenic, and other destructive effects on malignant cells in vitro and in vivo. Due to the complexity of these data and the great diversity of BP compounds, we will only overview some compounds that we have already highlighted during this work. Chemopreventive, cell death-inducing (mainly pro-apoptotic), antiproliferative, antimetastatic, and/or antiangiogenic effects were reported, for example, for the majority of BP flavonoids and phenolic acids that we enumerated in this publication (for review examples, see [391,392,393]). Moreover, many of these molecules have been reported to suppress cancer cell stemness and reduce malignant stem cell pools in experimental models (e.g., kaempferol [394], quercetin [395], genistein [396], and protocatechuic [397], ellagic [398], and caffeic [399] acids), while others have been shown to enhance antitumor immunity (e.g., apigenin [400], cyanidin-3-*O*-glucoside [401], delphinidin-3-*O*-glucoside [401], and chlorogenic acid [402]).

Carotenoids are other abundant BP compounds that are widely studied for their anticancer potential. Except for some limited controversies, mainly in lung cancer, carotenoids (including those that are present in BP, i.e., α-carotene, β-carotene, β-cryptoxanthin, lycopene, lutein, and zeaxanthin) have been endowed with chemopreventive, proapoptotic, and antimetastatic potential against different cancers in numerous studies in animal models and observational studies in humans [403,404,405].

In addition to polyphenols and carotenoids, many other BP compounds are known for their anticancer potential. Steroid compounds from *Brassica napus* and *Brassica campestris* showed important anticancer effects on prostate cancer [341]. Another study of BP of the plant *Lycium barbarum* reported that lipopolysaccharides from this pollen induced, in vitro and in vivo, a significant dose-dependent increase in cancerous cell apoptosis and tumor growth suppression by different mechanisms, including a particularly marked inhibition of the PI3K/Akt [406]. The latter is known to be one of the major signaling pathways that promote carcinogenesis, tumor growth and dissemination, and resistance to chemotherapeutic agents [407].

Phytosterols are also endowed with multifaceted preventive and pharmacological potentials against many cancers according to a considerable body of preclinical evidence. Mechanistically, they were reported to induce cancer cell death, exert anti-proliferative, antimetastatic, and antiangiogenic effects, stimulate antitumor immunity, and alter the tumor microenvironment [408,409,410]. Spermidine and its analogues have been shown to induce apoptosis and cell cycle arrest in diverse cancer cells [411]. In addition, some BP micronutrients are also widely known, at least from a nutritional point of view, to be related to cancer risk and progression. Anticancer properties were, for example, widely reported for vitamin E [412,413], polyunsaturated fatty acids [414,415], and selenium [416,417]. Regulatory authorities and scientific communities remain skeptical about the use of micronutrients for cancer prevention or treatment due to insufficient or unclear evidence, but studies of these nutrients in a holistic approach considering their natural existence milieus and association with other nutrients and phytochemicals, either from BP or other natural sources, are still lacking.

**Figure 4:**
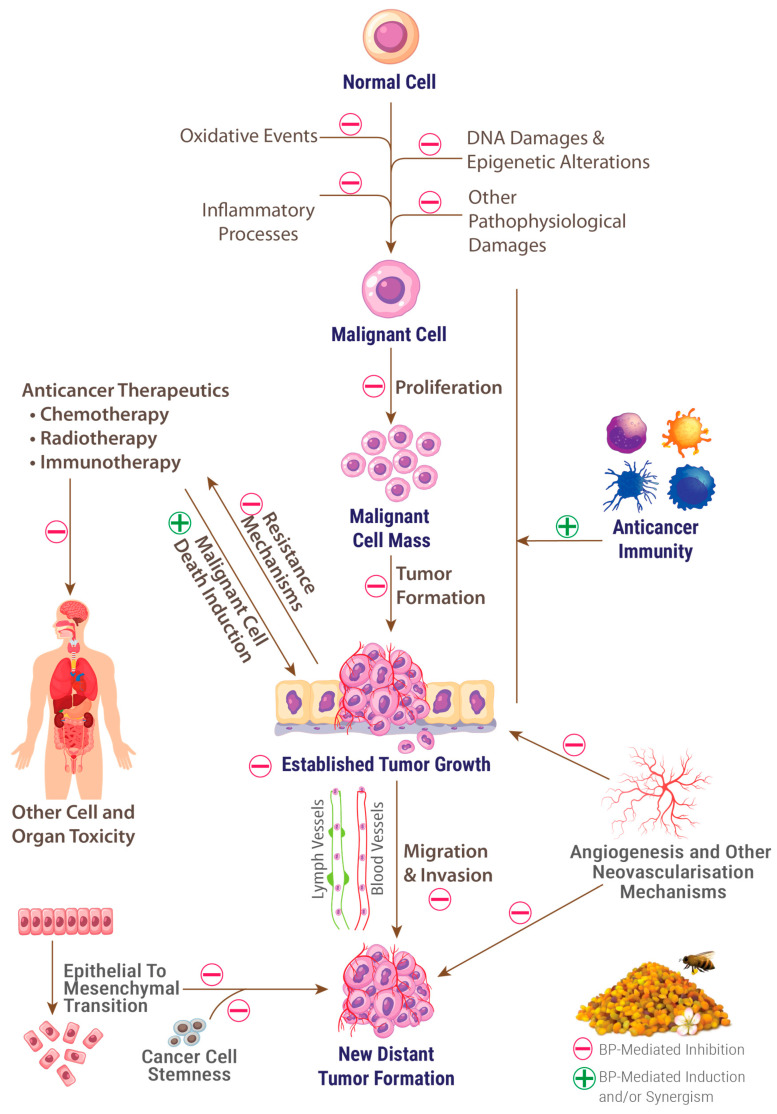
General Anticancer Mechanism of BP and Its Major Compounds.

All the major anticancer mechanisms of BP and its major compounds, reviewed in the current section, are summarized below in Figure 4.

## 6. Materials and Methods

### 6.1. Rationale

Human observational and interventional studies about BP in neurodegeneration and cancer are both still lacking. Likewise, studies investigating these topics in experimental animal models are also still very scarce. However, an immense amount of experimental evidence on different pathophysiological mechanisms that are involved in neurodegeneration and/or carcinogenesis is available on selected BP compounds and less abundantly on BP as a whole product or on its extracts. We have therefore estimated that a literature review is very needed. Furthermore, and given that the available experimental evidence is still heterogeneous and insufficiently clear despite its abundance, we have decided to adopt a scoping review methodology in our investigation.

### 6.2. Study Design

**Figure 5:**
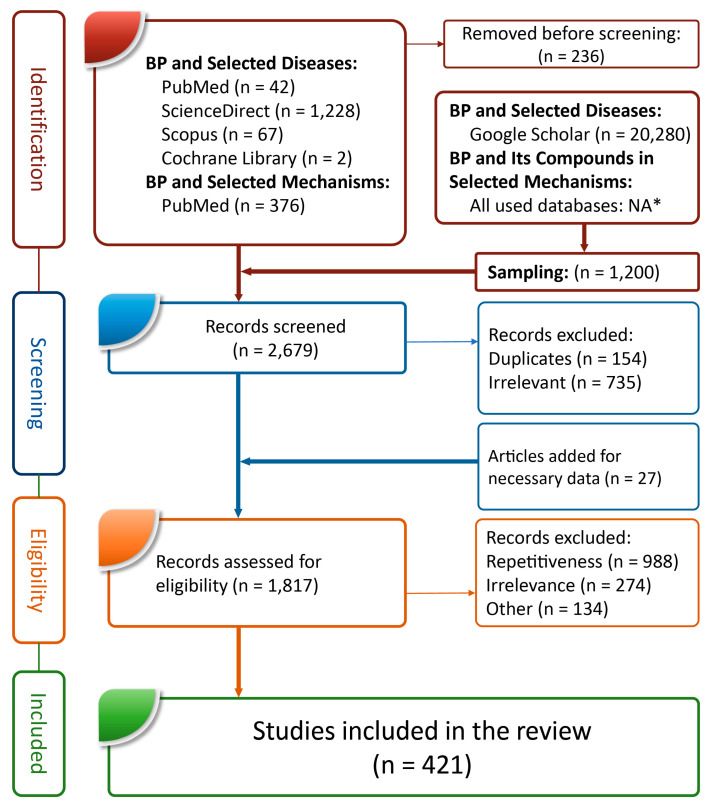
Flow Diagram of Literature Resource Identification and Screening (The protocol conception relies on PRISMA 2020 Standards, We have detailed it in our previous publication [11]). NA* (Not applicable): The sum of results in these databases were higher than 10,000, so we conducted a sampling of some studies depending on the biological actions that we searched (around 1200 articles were included in this sampling).

Scoping review is more adapted to the topic that we discuss in this work than other review methods. On the one hand, a scoping review permits one to expound a large extent of literature in a more flexible way that may include diversified and heterogeneous data. On the other hand, it ensures a systematic and comprehensive selection of available scientific literature while maintaining a greater openness to receive scientific community criticism and draw diversified conclusions [11]. In this context, scoping reviews are perfectly adapted to map available evidence on a complex and diversified product such as BP, especially in complex and insufficiently understood pathophysiological contexts such as cancers and neurodegeneration. In the current work, we adopt the same reference (PRISMA checklist and guidelines) that we adopted and explained in our recent review, which was, to our current knowledge, the first one that focused on BP [11]. Our review protocol is resumed in Figure 5, and the procedural checklist is presented in Table 2.

### 6.3. Search, Sampling, and Data Extraction

#### 6.3.1. Database Searches

The main international databases dealing with the medical and pharmaceutical fields, viz., PubMed, ScienceDirect, Scopus, Cochrane Library, and Google Scholar, were consulted. Two preliminary searches separately used the keywords “bee pollen cancer” and “bee pollen neurodegener*” to screen potentially relevant publications. In parallel, the mechanisms that are well studied and present in both neurodegeneration and cancer were identified.

#### 6.3.2. Pathophysiological Mechanism Selection

We selected oxidative stress, inflammation, cellular clearance, cell death, microbiota alterations, infections, and genetic and epigenetic alterations. For specific mechanisms, we selected neuroinflammation, enzyme modulation, and misfolded protein aggregation for neurodegenerative diseases. We focused only on the major neurotoxic proteins that are well documented in the most prevalent NDD, viz., amyloid-β, tau, and α-synuclein. In cancer we selected the two main issues in cancer therapies, viz. tumor suppression and therapy toxicity. In the tumor suppression, we searched major anticancer mechanisms, viz. cell death induction, inhibition of proliferation, metastasis and neovascularization, anticancer immunity, and carcinogenesis prevention, in addition to the synergism with anticancer therapeutics. To evaluate the role of BP on the toxicity of anticancer therapeutics, we searched for studies that covered the modulation of this toxicity by BP. In anticancer therapeutics, we considered all kinds, i.e., chemotherapy, radiotherapy, and immunotherapy. More affined terms were subsequently used, including “bee AND pollen” and one of the following words: “oxidation”, “inflammation”, “autophagy”, “apoptosis”, “ferroptosis”, “microbiota”, “bee DNA damage”, and “epigenetic”. The search term “bee pollen neurodegener*” was replaced by the “bee pollen neurodegenerative” in Science Direct because this database does not support searches with wildcard characters (“*” in our case). In this database, the “article type” filter was applied and limited to “review articles” and “research articles”.

#### 6.3.3. BP Compound Selection

After this first screening, we undertook a general phytochemical overview of BP studies and chose a list of the most frequent bioactive compounds and nutrients. We have chosen to investigate a series of the main reported compounds in BP as examples of diverse bioactivities that are tightly linked to cancer and neurodegeneration pathogenesis and therapeutic targeting. As examples of the most reported flavonoids in BP [22,23,27,30,32,37,43,53,56,247,418,419], we chose apigenin, catechin, chrysin, cyanidin, delphinidin, epicatechin, genistein, hesperidin, hesperetin, isorhamnetin, kaempferol, luteolin, myricetin, naringenin, naringin, pinocembrin, quercetin, and their respective glycosides. The main reported phenolic acids in BP [22,27,32,37,53,56,247,418], which we chose as examples to study, were benzoic, caffeic, chlorogenic, cinnamic, coumaric, dihydroxybenzoic, ellagic, ferulic, gallic, hydroxycinnamic, protocatechuic, rosmarinic, syringic, and vanillic acids. Although less frequently reported, we chose resveratrol as a relevant example of stilbene derivatives. The most frequent BP carotenoids [40,48,53,420,421] that we chose as study examples were α-carotene, β-carotene, β-cryptoxanthin, lutein, zeaxanthin, and lycopene. Other BP ubiquitous compounds that we studied as examples were spermidine and its glycosides among phenolamides, betaine and choline as relevant betaines, and glucosinolates, which we always reviewed as a chemical family without specific examples. Coenzyme Q10 as a BP compound with very relevant importance to the studied diseases was also reviewed. Among BP nutrients, we mainly focused on all vitamins and on copper, iron, selenium, and zinc as very relevant elements to neurodegeneration and cancer, in addition to phytosterols (mainly β-sitosterol as a major representative in BP [20,56,421]). The names of these compounds were therefore included to replace “bee pollen” in all the keyword searches that we have just enumerated. Other database consultations were continuously performed while advancing in our work to elucidate pathophysiological mechanisms or some properties of the studied BP compounds.

#### 6.3.4. Time Interval Selection

Due to the huge amount of data, we also decided to limit the time interval of includible literature. In the general anticancer and anti-neurodegenerative mechanisms of BP, we extended the search to the last ten-year interval, while in the case of BP-specific compounds, we limited the search to the last five years. The search for BP effects on specific mechanisms (e.g., oxidative, inflammation, autophagy, etc.) was also limited to the last five years. However, when data were unclear or insufficient, we extended the search on the issue concerned to larger time intervals. When studying disease pathophysiological parameters and related mechanistic issues, the search was limited to the last three years.

#### 6.3.5. Final Data Refinements

For the keyword “bee pollen” associated with “cancer” or “neurodegeneration”, a total of 42, 1228, 67, 2, and 20,280 articles were retrieved from PubMed, ScienceDirect, Scopus, Cochrane Library, and Google Scholar, respectively, from the last 10 years. For the keyword “bee pollen” associated with the keywords “oxidative”, “inflammation”, “autophagy”, “apoptosis”, “ferroptosis, “microbiota”, “DNA”, and “epigenetic”, the number of retrieved research works in PubMed were 116, 29, 4, 21, 0, 112, and 94, respectively. The number of results was large, with singular BP compounds, and a reference sampling was adapted to the investigated mechanism or the biological or pathophysiological process.

There was no screening of studies based on the intervention type, study type, or subject. All studies that were related to our chosen biological processes to investigate, including experimental, clinical, observational, reviews, and other studies, were considered. The selection of research publications was then limited to those published in English, French, and Spanish languages. The English language and a three-year limitation were used in Google Scholar to refine the search. From all initial search results, we have eliminated the following types from consideration: duplication across databases, books and conference proceedings, irrelevant publications for our research topic, and works that were published without a digital object identifier (DOI). The final number of articles that were included in the present review was 421.

### 6.4. Synthesis of Findings

Our current work was undertaken in a biphasic procedure. In addition to the scoping review to gather and map available evidence, we undertook an analytical approach to figure out possible research avenues to develop future investigational works in the tackled topic. Since the data about BP in neurodegeneration and cancer are roughly known to be encouraging, we adopted this approach concomitantly with reviewing the recent achievements in understanding the pathophysiological mechanisms for which we searched for a possible interest of BP and its known compounds. Such an approach consequently obligated us to analyze and report a great number of scientific publications, a number that is normally very uncommon in biomedical and pharmaceutical research regardless of the review type and topic.

## 7. Inferring Remarks and Perspectives

Following all that we have seen throughout this work, we can ascertain that BP is an unequaled stockpile of functional bioactive compounds and complementary nutraceuticals, which could be perfectly integrated into a healthy lifestyle for healthy persons and those with illnesses. This is especially relevant to carcinogenesis and neurodegeneration contexts where the major associated pathophysiological mechanisms are among the most important ones mitigated by BP. The spectrum of hallmarks of aging and age-related diseases that are widely accepted among the scientific community primarily includes genomic and epigenetic alterations, loss of protein homeostasis, impaired autophagy, mitochondrial dysfunction, cellular senescence, compromised neuronal function and plasticity, chronic inflammation, immunosenescence, dysbiosis, and deregulated nutrientsensing [223,312]. We have thoroughly elucidated in this work, as well as in our recent publication [11], that BP and its known compounds act through a multitude of mechanisms in most of these hallmarks.

In all the mechanisms discussed, the chronicity of resulting modifications is a common attribute preceding the settlement of both neurodegenerative and malignant disorders. Effective mitigation of such deleterious results requires a safe and holistically rich tool to suppress chronic triggers. BP then emerges as a candidate encompassing a wide range of verified bioactivities that could be a valuable contribution either in disease prevention or in certain stages of disease progression. However, the number of preclinical and clinical studies remain very limited given the extensive diversity of BP and the great diversity, ambiguity, and entanglement of carcinogenesis- and neurodegeneration-related processes.

In our current work, we present, for the first time to our knowledge, substantial and clear evidence coming from individual studies of a wide range of BP compounds supporting the existence of additional, very relevant bioactivities of BP that remain completely untapped. These bioactivities are mainly the modulation of ferroptosis and the regulation of epigenetic mechanisms.

Most studies investigating natural, especially phenolic, compounds in ferroptosis as it relates to neurodegeneration and cancer were published in the last three to five years, depending on the molecules. Data is still raw, and, despite the promising results that emanate from preclinical evidence, our understanding of these effects remains elementary, and further research works are needed to achieve beneficial clinical outcomes. BP is packed with diverse ferroptosis modulators that seem to act in intricate ways depending on the cellular context. Although a great part of the compounds studied exhibit a bimodal ferroptosis regulation for the same compound, many others have been reported to only mitigate ferroptosis. These intriguing qualities undeniably warrant further substantial research efforts. The ferroptosis inhibitory effect of its compounds may endow BP with a significant interest in mitigating neurodegeneration, a process that develops through decades of alterations and culminates in cell death that appears to be driven by ferroptosis in many NDDs. This does not alter its potential in managing cell death also in carcinogenesis due to numerous examples that we have seen. In fact, aggressive, metastatic, and treatment-refractory cancer cells are known to be highly sensitive to ferroptosis. Figuring out safe and chronically usable compounds, such as BP, is thus of paramount importance.

On the other hand, the plethora of epigenetic regulatory mechanisms that we have seen for many major BP compounds is clearly relevant. Epigenetic regulation is a very promising research area in BP, and a highly complex and multifaceted potential is a priori verified for a wide spectrum of BP compounds. Since epigenetic processes are among the most studied and impactful etiological factors in neurodegeneration, cancers, and age-related diseases in general, more focused studies are strongly recommended to decipher possible interventions, especially in long-term preventive interventions, but also in managing confirmed pathological states and/or in conducting prenatal interventions.

The other promising research avenue is the modulation of microbiota. The latter has been investigated by a few experimental studies of BPs and by a wide range of studies that explored individual BP compounds, but investigations focusing on cancer and neurodegeneration are still insufficient, especially for BP as a whole product or for its extracts. We will soon develop this topic in a separate publication.

The fourth research avenue which may encompass great importance for BP is the mitigation of infections and other disorders that accompany them, particularly given their roles in increasing risks to develop oncological and neurodegenerative disorders or to exacerbate them through promoting comorbidities.

The fifth research topic that remains untapped by BP research is the potential of this bee-fashioned cocktail to mitigate aberrant protein aggregation and the neurotoxicity that is mediated by these misfolded proteins. We have also clearly exposed the abundance of experimental evidence emanating from individual studies of BP compounds in this topic.

Other specific topics were also evoked during our discussions. Among appealing examples, we underscore the potential role of BP polysaccharides in ferroptosis, and the calorie restriction mimetic that was unveiled for some BP compounds.

We must underline that our current work has some limitations. The first and most important one is the insufficient understanding of the pathophysiological mechanisms, especially in the case of neurodegeneration which may have influenced the definition of the pathophysiological processes to focus on in the current study. The second limitation was the quasi-absence of the coordination and officially agreed standardization of experimental studies of BP, resulting frequently in scattered results and insufficient approaches of the main studied bioactivities. The third limitation was the great scarcity in some major pathophysiological processes in neurodegeneration for BP as a whole product, especially in the areas that we proposed to investigate for the first time such as epigenetic regulation and ferroptosis modulation. The fourth major limitation was the absence of the studies evaluating the interaction between different BP compounds, either in synergistic or antagonistic manners, in the different bioactivities that we have studied in this review. Other minor limitations, such as the impossibility to access some publications and some clearly inconsistent results reported by some experimental studies, were encountered but did not significantly influence the essence of our review, analysis, and inferences.

We could index all the novel research avenues we have proposed as the first major outcome of our investigation. The second outcome was the identification of diverse challenges that may still hinder the application of the encouraging experimental evidence in translational research and clinical practice.

One primary challenge to address is the problem of bioavailability and bioaccessibility of BP bioactive compounds. As is well known in natural product pharmacology, some very promising compounds of BP still could not be efficiently delivered in situ to the desired targets throughout the body. Most aspects of gastrointestinal and systemic metabolism, as well as overall pharmacokinetic and pharmacodynamic behaviors of these compounds, remain poorly understood. Additionally, some drawbacks, such as great instability and short half-life, that characterize some phenolics and other BP compounds remain unsolved.

The second most important challenge is the standardization of BP composition. Unfortunately, the establishment of agricultural, apicultural, and experimental guidelines to conceive reproducible BP composition, as well as reliable analytical procedures and efficient formulations remains still far from our reach. Another great deficiency that persists in understanding BP is the rarity of comparative studies between the phytochemical composition of plant pollens in flowers and that of BPs. Comparative analyses of pharmacokinetic and pharmacodynamic traits of both pollens are also still missing.

A final important parameter to consider is the fact that multitargeting is a highly coveted tendency in pharmacological prospection regarding complex and poorly understood diseases such as NDD and cancers. In the case of BP, this can be either an advantage or a limitation, depending on the investigational and clinical contexts. The synergistic and eventual antagonistic behaviors among BP compounds in bioactivities that are relevant for neurodegeneration and cancer remain almost absent.

## 8. Conclusions

BP is a rich natural source of biopharmaceuticals that are endowed with a wide spectrum of bioactivities on nearly all the known major pathophysiological processes that participate in neurodegeneration and cancer pathogenesis. The multiple, encouraging, and promising bioactivities and insights that we have detailed in this work lead us to firmly bolster, not only the preventive, but also the possible disease-modifying potential of BP in neurodegeneration and cancer. This work is the first comprehensive review of BP potential in neurodegenerative and malignant disorders. We hope that it will provide motivating insights to address the research issues we have raised and will serve as valuable starting point for future, more impactful scientific works.

## Figures and Tables

**Table 2:** Research Protocol According to the Reporting Guidance of PARISMA-ScR Checklist for Scoping Reviews and Updated Recommendations from PRISMA 2020 Statement for Systematic Reviews.

SECTION	ITEM	PRISMA-ScR CHECKLIST ITEM	REPORTED ON PAGE #
**TITLE**			
**Title**	1	Identify the report as a scoping review.	1
**ABSTRACT**			
**Structured summary**	2	Provide a structured summary that includes (as applicable): background, objectives, eligibility criteria, sources of evidence, charting methods, results, and conclusions that relate to the review questions and objectives.	1
**INTRODUCTION**			
**Rationale**	3	Describe the rationale for the review in the context of what is already known. Explain why the review questions/objectives lend themselves to a scoping review approach.	1, 2, 3
**Objectives**	4	Provide an explicit statement of the questions and objectives being addressed with reference to their key elements (e.g., population or participants, concepts, and context) or other relevant key elements used to conceptualize the review questions and/or objectives.	1, 2, 3, 25
**METHODS**			
**Protocol and registration**	5	Indicate whether a review protocol exists; state if and where it can be accessed (e.g., a web address); and if available, provide registration information, including the registration number.	None
**Eligibility criteria**	6	Specify characteristics of the sources of evidence used as eligibility criteria (e.g., years considered, language, and publication status), and provide a rationale.	25, 26
**Information sources**	7	Describe all information sources in the search (e.g., databases with dates of coverage and contact with authors to identify additional sources), as well as the date the most recent search was executed.	25, 26
**Search**	8	Present the full electronic search strategy for at least 1 database, including any limits used, such that it could be repeated.	25, 26
**Selection of sources of evidence**	9	State the process for selecting sources of evidence (i.e., screening and eligibility) included in the scoping review.	25, 26
**Data charting process**	10	Describe the methods of charting data from the included sources of evidence (e.g., calibrated forms or forms that have been tested by the team before their use, and whether data charting was completed independently or in duplicate) and any processes for obtaining and confirming data from investigators.	26, 27
**Data items**	11	List and define all variables for which data were sought and any assumptions and simplifications made.	25–27
**Critical appraisal of individual sources of evidence**	12	If completed, provide a rationale for conducting a critical appraisal of included sources of evidence; describe the methods used and how this information was used in any data synthesis (if appropriate).	None
**Synthesis of results**	13	Describe the methods of handling and summarizing the data that were charted.	26, 27
**RESULTS**			
**Selection of sources of evidence**	14	Give numbers of sources of evidence screened, assessed for eligibility, and included in the review, with reasons for exclusions at each stage, ideally using a flow diagram.	27
**Characteristics of sources of evidence**	15	For each source of evidence, present characteristics for which data were charted and provide the citations.	5–24
**Critical appraisal within sources of evidence**	16	If completed, present data on critical appraisal of included sources of evidence (see item 12).	None
**Results of individual sources of evidence**	17	For each included source of evidence, present the relevant data that were charted that relate to the review questions and objectives.	3–24
**Synthesis of results**	18	Summarize and/or present the charting results as they relate to the review questions and objectives.	5–24, 30–31
**DISCUSSION**			
**Summary of evidence**	19	Summarize the main results (including an overview of concepts, themes, and types of evidence available), link to the review questions and objectives, and consider the relevance to key groups.	3–24
**Limitations**	20	Discuss the limitations of the scoping review process.	31
**Conclusions**	21	Provide a general interpretation of the results with respect to the review questions and objectives, as well as potential implications and/or next steps.	30–31
**FUNDING**			
**Funding**	22	Describe sources of funding for the included sources of evidence, as well as sources of funding for the scoping review. Describe the role of the funders of the scoping review.	32

Table template according to the updated recommendations from the PRISMA 2020 statement were retrieved from the updated version of the JBI Manual for Evidence Synthesis (https://jbi-global-wiki.refined.site/space/MANUAL/355862497) (accessed on 19 November 2024). Methodological details could be found in our recent previous publication [11].
